# Dietary approaches to treat MS-related fatigue: comparing the modified Paleolithic (Wahls Elimination) and low saturated fat (Swank) diets on perceived fatigue in persons with relapsing-remitting multiple sclerosis: study protocol for a randomized controlled trial

**DOI:** 10.1186/s13063-018-2680-x

**Published:** 2018-06-04

**Authors:** Terry Wahls, Maria O. Scott, Zaidoon Alshare, Linda Rubenstein, Warren Darling, Lucas Carr, Karen Smith, Catherine A. Chenard, Nicholas LaRocca, Linda Snetselaar

**Affiliations:** 1 University of Iowa, Iowa City, Iowa, USA; 2 National Multiple Sclerosis Society, New York, New York, USA

**Keywords:** Multiple sclerosis, Fatigue, Diet, Accelerometer, Quality of life, Intervention, Swank diet, Wahls elimination diet

## Abstract

**Background:**

Fatigue is one of the most disabling symptoms of multiple sclerosis (MS) and contributes to diminishing quality of life. Although currently available interventions have had limited success in relieving MS-related fatigue, clinically significant reductions in perceived fatigue severity have been reported in a multimodal intervention pilot study that included a Paleolithic diet in addition to stress reduction, exercise, and electrical muscle stimulation. An optimal dietary approach to reducing MS-related fatigue has not been identified. To establish the specific effects of diet on MS symptoms, this study focuses on diet only instead of the previously tested multimodal intervention by comparing the effectiveness of two dietary patterns for the treatment of MS-related fatigue. The purpose of this study is to determine the impact of a modified Paleolithic and low saturated fat diet on perceived fatigue (primary outcome), cognitive and motor symptoms, and quality of life in persons with relapsing-remitting multiple sclerosis (RRMS).

**Methods/design:**

This 36-week randomized clinical trial consists of three 12-week periods during which assessments of perceived fatigue, quality of life, motor and cognitive function, physical activity and sleep, diet quality, and social support for eating will be collected. The three 12-week periods will consist of the following:

1. *Observation*: Participants continue eating their usual diet.
2. *Intervention*: Participants will be randomized to a modified Paleolithic or low saturated fat diet for the intervention period. Participants will receive support from a registered dietitian (RD) through in-person coaching, telephone calls, and emails.
3. *Follow-up*: Participants will continue the study diet for an additional 12 weeks with minimal RD support to assess the ability of the participants to sustain the study diet on their own.

**Discussion:**

Because fatigue is one of the most common and disabling symptoms of MS, effective management and reduction of MS-related fatigue has the potential to increase quality of life in this population. The results of this study will add to the evidence base for providing dietary recommendations to treat MS-related fatigue and other symptoms associated with this disease.

**Trial registration:**

ClinicalTrials.gov, NCT02914964. Registered on 24 August 2016.

**Electronic supplementary material:**

The online version of this article (10.1186/s13063-018-2680-x) contains supplementary material, which is available to authorized users.

## Background

Fatigue is one of the most common and disabling symptoms of multiple sclerosis (MS), diminishing quality of life (QOL) and contributing to early exit from the workforce [1, 2]. MS-related fatigue is most commonly managed through multiple interventions, including disease-modifying drugs and stimulants, exercise, energy conservation, and stress management techniques [3]. Although exercise augmented by electrical muscle stimulation can be modestly effective in reducing perceived fatigue [4, 5], studies investigating the efficacy of pharmaceutical therapies have shown conflicting results [6–8]. Because drug treatment has not been effective, dietary interventions are being explored. Statistically and clinically significant reductions in perceived fatigue severity in persons with progressive multiple sclerosis (pwPMS) have been reported with use of a multimodal intervention consisting of a modified Paleolithic diet, stress reduction, exercise, and electrical muscle stimulation [4, 5].

Interventions considering the whole diet (vs. supplement-based, single-nutrient focus) have been used in treating or preventing diseases, including psoriasis [9], cancer [10, 11], and neurological diseases [12]. Emerging data support the notion that environmental rather than genetic factors are likely the predominant causes of MS [13]. Given that food consumed is a major component of the environment, it is conceivable that improving the quality of the diet may have a significant impact on the development of MS. The relationship between the quality of the diet and MS-related symptoms such as fatigue is unknown. In this study, we will compare two dietary patterns for the treatment of MS-related fatigue: the modified Paleolithic diet (Wahls elimination diet) and a low saturated fat diet (Swank diet).

One early dietary intervention for individuals with MS was based on the observation that high levels of saturated fat in the diet were associated with increased risk for MS in Norway [14, 15]. Dr. Roy Swank theorized that a diet high in saturated fats causes more rapid disease progression. Dr. Swank followed 144 patients with mild to more severe disability for 34 years. These individuals had agreed to consume a diet containing < 20 g of saturated fat per day and report their dietary adherence. The patients’ clinical outcomes were monitored, including physical and mental performance [16–21]. The Swank study found that the number of relapses and progression of disability was associated strongly with dietary saturated fat consumption [17–22]. The 50-year follow-up is a strength of the Swank study, but the absence of a control group and lack of brain imaging are limitations.

Consumption of vegetables has also been associated with favorable health outcomes related to MS. Notably, the mean daily serving of vegetables is associated with lower risk of developing obesity [23], which is a risk factor for and a common comorbid diagnosis of those with MS. Increased consumption of vegetables is associated with lower Expanded Disability Status Scale scores [24], insulin sensitivity, blood pressure, body weight, and body mass index (BMI). Considering these observations, researchers in a more recent randomized controlled trial used a vegetarian version of the Swank diet [25], also known as the McDougall diet. Measures included the Fatigue Severity Scale (FSS), 36-item Short Form Health Survey (SF-36) quality-of-life scores, lipids, weight, BMI, and brain magnetic resonance imaging (MRI) scans at baseline and at 1 year [26]. Favorable reductions in weight, BMI, and total cholesterol were observed, but no statistically significant differences in MRI findings or SF-36 quality-of-life scores were reported [26].

Another diet of interest to the MS community is a Paleolithic diet [27]. Dr. Loren Cordain’s recommendations for a modern version of the Paleolithic diet stresses the consumption of meats, vegetables, and fruits; excludes grains, legumes, and dairy [27, 28]; and excludes nightshade vegetables (potatoes, tomatoes, peppers, and eggplants) [29] for persons with rheumatoid arthritis. Recently tested for its impact on various biomarkers in healthy individuals, the Paleolithic diet was associated with improvements in blood pressure, BMI [30], total cholesterol, insulin sensitivity, fasting insulin, and arterial distensibility [31]. In a study of patients with type 2 diabetes, the Paleolithic diet was shown to be more satiating per calorie than the American Diabetes Association (ADA) diet, which encourages increased intake of vegetables, dietary fiber, whole-grain bread and cereal products, fruits, and berries and decreased intake of total fat with more unsaturated fat [32]. In a crossover study comparing the Paleolithic diet with the ADA diet, the Paleolithic diet was superior to the ADA diet with respect to improving blood pressure, lipid profile, and glycemic control [33]. Finally, in a randomized controlled study of obese persons with metabolic syndrome, comparison of the Paleolithic diet with the control diet, which was an isoenergetic diet based on Dutch dietary guidelines, the Paleolithic diet was associated with greater improvements in blood pressure, fasting levels of lipids, and weight loss than the control diet [34].

A modified version of the Paleolithic diet was shown to reduce perceived fatigue in pwPMS (either secondary or primary progressive multiple sclerosis [SPMS or PPMS, respectively]) [4, 5] as part of a multimodal intervention (modified Paleolithic diet, targeted vitamin supplementation, stress-reducing practices, exercise, and electrical muscle stimulation). The study diet stressed the consumption of more vegetables, with a target of 6 to 9 cups of vegetables and fruit per day, and recommended somewhat less meat than Paleolithic diets tested in the previously mentioned studies. At enrollment, study participants were consuming less than 1.5 servings of vegetables per day but raised this to an average of 8 servings per day by month 12 [5]. The dietary component of the multimodal intervention was significantly associated with favorable changes in mood and cognition between baseline and 12 months, whereas the nondietary components were not [25]. It is unknown whether the dietary component of the multimodal intervention also significantly contributed to the observed reduction in perceived fatigue [4, 5]; however, several participants anecdotally reported that deviations from the study diet resulted in a sharp worsening of their fatigue and noted that the fatigue resolved with stricter adherence to the study diet. Data from another pilot randomized controlled trial also showed significant reductions in perceived fatigue (as assessed by FSS) in individuals with relapsing-remitting multiple sclerosis (RRMS) following a modified Paleolithic diet intervention [35].

To establish the specific effects of diet on MS symptoms such as fatigue, this study focuses on diet only instead of the previously tested multimodal intervention. The modified Paleolithic diet continues to stress a high intake of vegetables but also eliminates foods to which some individuals may be sensitive: eggs and nightshade vegetables [29]. To enhance adherence and reduce the rate of dropout, which occurred early in the intervention among participants in a nondiet control group [35], control participants will be assigned a second diet, a low saturated fat (Swank) diet, which is also popular among the MS community and has research to support its efficacy.

## Methods/design

### Aims of study

In this study, we will compare the impact of the modified Paleolithic diet (Wahls elimination diet) and a low saturated fat diet (Swank diet) on perceived fatigue, cognitive and motor symptoms, and quality of life in persons with RRMS, a milder form of disease that often transitions to a more progressive and severe form of MS. We hypothesize that participants following the modified Paleolithic diet will have a clinically greater reduction in FSS score than participants following the low saturated fat diet.

#### Research hypothesis 1a

After 12 weeks of the diet intervention, relative to the observation period, at least 15% of participants in the low saturated fat diet group will demonstrate decreased perceived fatigue (assessed by FSS score), 30% will have improvement in QOL mental health and physical health scores (mean score increase of > 5 points), 50% will demonstrate improved cognition (> 5% improvement in mean Symbol Digit Modalities Test–Oral [SDMT-O] score), and 30% will demonstrate improved motor function (> 5% improvement in mean distance of 6 Minute Walk Test [6MWT]). In the modified Paleolithic diet group, ≥ 60% of the participants will demonstrate decreased perceived fatigue, 70% will have improvement in QOL mental health and physical health score, 70% will demonstrate improved cognition, and 55% will demonstrate improved motor function.

#### Research hypothesis 1b

After 12 weeks of the diet intervention, participants in the low saturated fat diet group will not have clinically or statistically significant reductions in fatigue or improvements in QOL measures or motor and cognitive function relative to the observation period, whereas participants in the modified Paleolithic diet group will have clinically and statistically significantly reduced fatigue and improved QOL measures and motor and cognitive function as measured by changes in mean scores of the aforementioned measures.

#### Research hypothesis 2a

By 12 weeks after initiation of diet, participants in the modified Paleolithic diet group will have a clinically greater reduction, relative to the observation period, of perceived fatigue (assessed by FSS) than those in the low saturated fat diet group.

#### Research hypothesis 2b

By 12 weeks after initiation of diet, participants in the modified Paleolithic diet group will have clinically greater improvement, relative to the observation period, with respect to both motor function (gait, assessed by the timed 25-ft walk test [T25FW] and by the 6MWT; hand, assessed by 9-hole peg test (9HPT); physical activity outcomes, measured by accelerometer [e.g., increased daily steps, increased time spent in light-intensity activity, increased time spent in moderate-intensity activity, reduced time spent sedentary]; sleep quantity, as measured by accelerometer) and cognitive function (assessed by the SDMT-O) than those in the low saturated fat diet group.

#### Research hypothesis 2c

By 12 weeks after the initiation of diet, participants in the modified Paleolithic diet group will have clinically greater improvement, relative to the observation period, with respect to quality of life (assessed by Multiple Sclerosis Quality of Life 54 [MSQOL54] instrument) and mood (assessed by Hospital Anxiety and Depression Scale [HADS]) than those in the low saturated fat diet group.

#### Research hypothesis 3

After 24 weeks of the diet intervention, changes in the measures identified in hypotheses 1a and 1b observed at 12 weeks will be sustained in ≥ 80% of the participants in both diet groups.

### Study Design

This 36-week randomized parallel group clinical trial consists of three 12-week periods during which assessments of perceived fatigue, quality of life, motor and cognitive function, physical activity and sleep, diet quality, and social support for eating will be collected. The 12-week periods will consist of:

1. *Observation*: Participants continue eating their usual diet.
2. *Intervention*: Participants will be randomized to either the modified Paleolithic or low saturated fat diet for the intervention period. Participants will receive support by a registered dietitian (RD) through coaching telephone calls and emails.
3. *Follow-up*: Participants will continue the study diet for an additional 12 weeks with minimal RD support to assess the ability of the participant to sustain the study diet on their own.

**Fig. 1:**
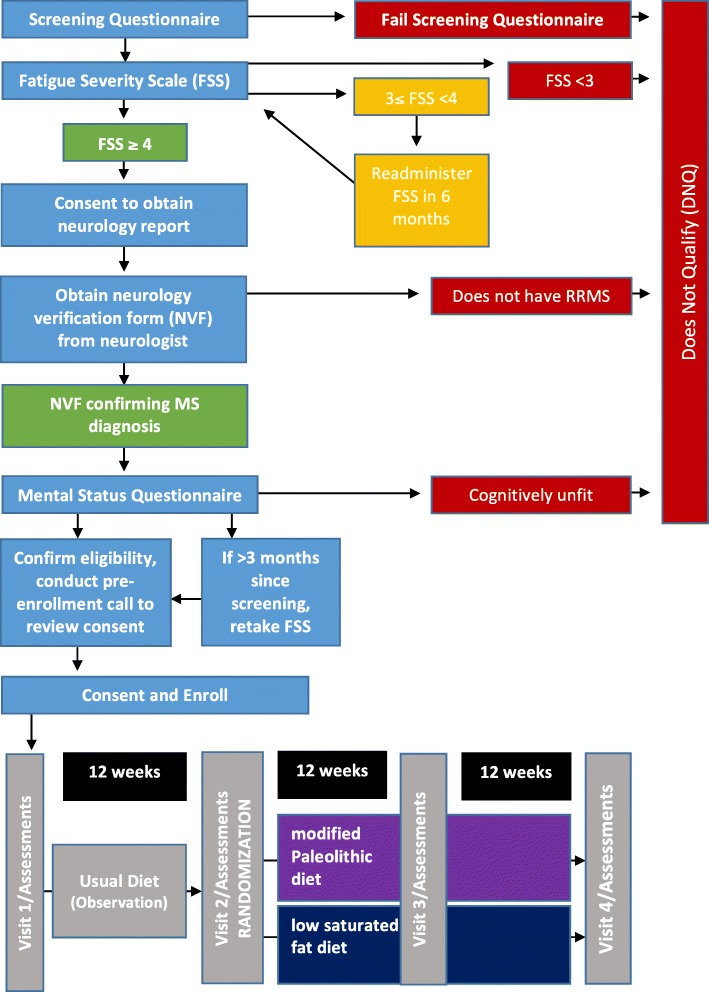
Dietary Approaches to Treat Multiple Sclerosis-Related Fatigue Study flowchart. *MS* Multiple sclerosis *RRMS* Relapsing-remitting multiple sclerosis

**Fig. 2:**
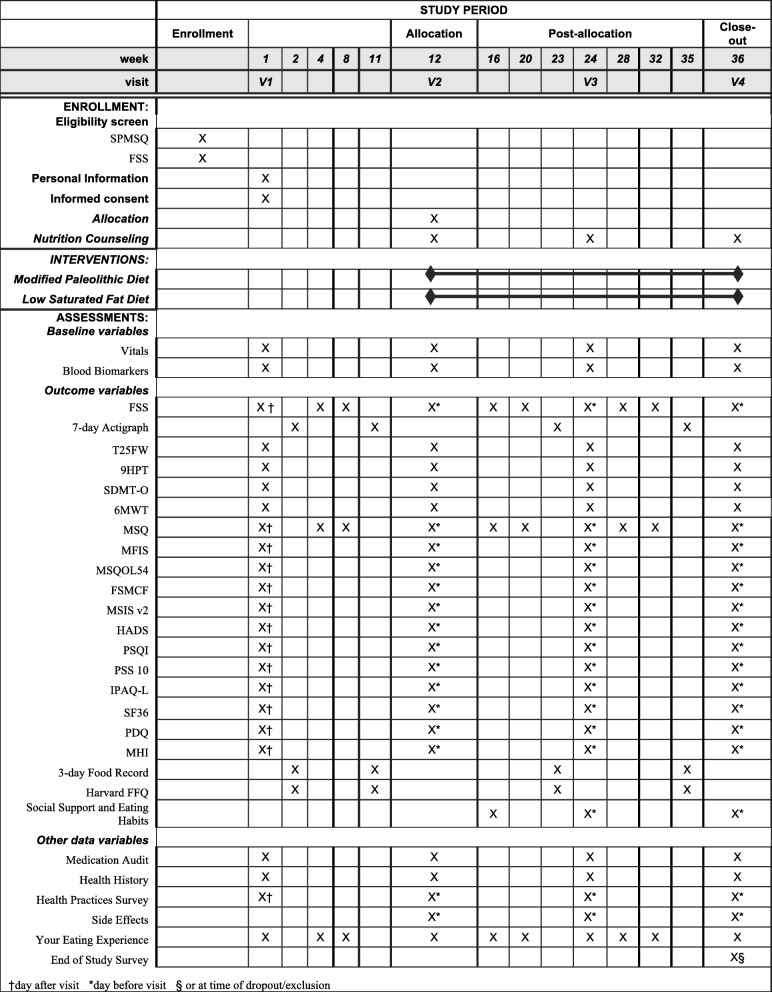
Standard Protocol Items: Recommendation for Interventional Trials (SPIRIT): the schedule of enrollment, interventions, and assessments. *SPMSQ* Short Portable Mental Status Questionnaire, *FSS* Fatigue Severity Scale, *9HPT* 9-Hole Peg Test, *T25FW* Timed 25-foot walk, *SDMT-O* Symbol Digit Modalities Test–Oral, *6MWT* 6-Minute walk test, *MSQ* Medical Symptoms Questionnaire, *MFIS* Modified Fatigue Impact Scale, *MSQOL54* Multiple Sclerosis Quality of Life 54, *FSMCF* Fatigue Scale for Motor and Cognitive Function, *MSIS v2* Multiple Sclerosis Impact Scale version 2, HADS Hospital Anxiety and Depression Scale, PSQI, *PSS 10* Perceived Stress Scale 10, *IPAQ-L* International Physical Activity Questionnaire–Long, SF36 36-Item Short Form Health Survey, *PDQ* Perceived Deficits Questionnaire, *MHI* Mental Health Inventory, FFQ Food Frequency Questionnaire

Study evaluations will be done to compare the effects of the two study diets on perceived fatigue (primary outcome measured by FSS) and secondary outcomes (cognitive function, motor function, QOL). The protocol was prepared according to the Standard Protocol Items: Recommendations for Interventional Trials (SPIRIT) guidelines (*see* Figs. 1 and 2 and Additional file 1: SPIRIT Checklist).

### Study setting

Data collection will be completed at the University of Iowa (UI) Preventive Intervention Center in Iowa City, IA, USA, by trained research assistants blinded to the group assignment. The medical monitor will be blinded to group assignment. Unblinding of assessors and medical monitors will not be permitted. To ensure that the proposed study is conducted without bias toward either of the study diets, Dr. Wahls will not have access to study participants or raw study data and will not be involved in data analysis.

### Eligibility criteria

Adults with RRMS who can ambulate 25 ft with either no support or unilateral support, living within approximately a 500-mile radius of Iowa City, meeting eligibility criteria, and providing written informed consent will be invited to participate. The inclusion criteria are outlined below:

1. a. Willing to allow their neurologist to sign a letter confirming MS diagnosis and criteria used to confirm diagnosis
  b. Definitive diagnosis of RRMS based on the revised 2010 McDonald criteria [36], confirmed by the treating neurologist
2. FSS score ≥ 4 obtained within 12 weeks of scheduling their first study visit (Screened participants with FSS ≥ 3 and < 4 will be contacted again in 6 months to retake FSS to determine if they qualify, if participants desire.)
3. Ability to shop for and prepare, or availability of someone in the family to shop for and prepare, home-cooked meals according to the study diet guidelines
4. Willing to keep detailed food records of all consumed foods and beverages
5. Aged between 18 and 70 years at time of enrollment into the study
6. Willing to eat a diet that includes more vegetables and excludes many comfort foods such as those made with white flour
7. Willing to eat a diet that eliminates red meat (beef, pork, lamb, veal), limits saturated fats (butter, coconut oil, margarine, and hydrogenated oils found in processed foods) to 15 g per day, and includes unsaturated fats (e.g., vegetable oils, nuts, fatty fish) to 20–50 g (4–10 teaspoons) per day if assigned to the low saturated fat diet but willing to eat saturated fats > 20 g per day if assigned to the modified Paleolithic diet
8. Not pregnant or planning to become pregnant in the next 12 months
9. Able to walk 25 ft without support or with only unilateral support (i.e., cane in one hand)
10. Have not been told by a physician or other health care professional that they have celiac disease
11. Willing and able to eat gluten-containing grains
12. Lives within approximately a 500-mile radius of Iowa City, which includes Illinois, Indiana, Iowa, Kansas, Michigan, Minnesota, Missouri, Nebraska, Ohio, South Dakota, and Wisconsin

The exclusion criteria are outlined below:

1. Taking insulin or warfarin
2. Having a relapse during the prior 12 weeks before consenting (The screening process will continue, with screening scheduled at least 12 weeks after the last relapse start date, if relapse happened after consenting; participation will be continued.)
3. Undergoing treatment for cancer by radiation or chemotherapy within the prior 12 months other than for skin cancer (They will be contacted after the 12 months if participants desire.)
4. Being diagnosed with kidney stones, heart failure, angina, or liver cirrhosis
5. Having a psychiatric disease, such as schizophrenia, that makes study adherence more difficult (Participants with depression and anxiety diagnoses are allowed.)
6. Having a diagnosis of an eating disorder such as anorexia, bulimia, binge eating, or orthorexia
7. Having BMI < 19 kg/m^2^
8. Having a moderate to severe mental impairment as measured by the Short Portable Mental Status Questionnaire (SPMSQ) [37]
9. Participating in another research study investigating MS or other medications, diet, supplements, exercise, or other treatments
10. Having had gastric bypass surgery with their treating physician believing it is medically unsafe to participate in this study
11. Having been told by a physician or other health care provider they have celiac disease
12. Have adverse physical reaction to eating gluten-containing products
13. Living outside a 500-mile radius of Iowa City
14. Not able or willing to comply with the study protocol

Exclusion criteria for continuation into the intervention phase of the study are as follows:

1. Inability to obtain blood at visit 1 or 2
2. Not providing a sufficiently detailed visit 1 food record
3. Not mailing back visit 1 study materials by study day 28
4. Not responding to queries for clarification of food record details within 7 days of the second attempt to contact

### Recruitment

Participants will be recruited from within a 500-mile radius of Iowa City. The North Central Multiple Sclerosis Society Region, which serves Iowa and Nebraska, has over 8000 members. The research team will work with the support groups of the National Multiple Sclerosis Society (NMSS) in communities within a 2-hour driving distance of Iowa City to advertise the study to their members. Additionally, the study team will work with regional MS centers, the North American Research Committee on Multiple Sclerosis (NARCOMS), the UI Hospitals neurology clinic, the Iowa City VA neurology clinic, the Swank Foundation, terrywahls.com, and other organizations to recruit study participants.

### Randomization

Participants will be randomized to the modified Paleolithic or low saturated fat diet on the basis of the screening FSS score that qualified them for the study. Two randomization tables based on computer software will be used, one for moderate perceived fatigue (a score ≥4 and < 5.5) and the other for high perceived fatigue (≥ 5.5), so that similar mean FSS scores are achieved for each diet group. The randomization tables are accessible (password-protected) only by intervention dietitians.

### Intervention

#### Study diets

The study diets are summarized in Table 1.

**Table 1:** Study diets

	Modified Paleolithic (Wahls Elimination Diet)	Low Saturated Fat (Swank Diet)
Recommended	• 2–3 cups (6 cups raw) leafy greens/day • 2–3 cups sulfur rich vegetables/day • 2–3 cups colorful fruits/vegetables/day • 6–12 oz. meat, fish, poultry or game/day • Approved fats (Extra virgin olive oil, coconut oil/butter, ghee, avocado, flax, hemp, walnut, sesame and sunflower oil, tahini, rendered animal fats and bacon grease)	• 2 cups vegetables/day • 2 cups fruit/day (fresh preferred) • 2 cups fat-free dairy/day • 4 servings grain and cereal/day (whole preferred) • 4 oz. + skinless chicken/turkey breast, white fish or shellfish/day • 4–10 tsp. (20–50 g) unsaturated fat/day
Encouraged or Limited	• 12 oz. organ meat/week • 16 oz. omega-3 fish/week • 1 serving seaweed, algae, nutritional yeast/day • 1 serving fermented food/day • Maximum 4 oz. nuts and seeds/day (soaked preferred) • Maximum 2 tbsp. flax, hemp and/or walnut oil/day • Flax and chia seeds to achieve 2 soft bowel movements/day • Maximum 1 alcoholic beverage/day (women) • Maximum 2 alcoholic beverages/day (men) • Maximum 1 tsp. approved sweetener/day (honey, molasses, maple syrup, stevia or sugar)	• Maximum 3 egg yolks/week • Maximum 3 cups caffeinated beverages/day • Maximum 1 alcoholic beverage/day • Maximum 15 g saturated fat/day • Maximum 50 g unsaturated fat/day
Not Recommended	• Dairy products (cow, goat, mare, soy milk, rice milk) • Grains with gluten • Gluten-free grains • Eggs • Legumes (beans, peas, soy, lentils, peanuts) • Nonallowed sweeteners; approved sweeteners > 1 tsp. per day • Nightshade vegetables/spices during first 12 weeks on diet • White fruits/vegetables (apple, pear, white potato) • Vegetable oils (corn, soy, canola, palm kernel, cottonseed, grapeseed), partially hydrogenated • Monosodium glutamate • Irradiated, deep fried, or microwaved food	• Red meat (beef, pork, lamb, veal, liver, kidney, heart, tongue) • Chicken or turkey dark meat • Rabbit, venison, elk, squab, pheasant • Dairy products with > 1 g saturated fat per serving • Foods containing hydrogenated fat or > 1 g saturated fat per serving such as desserts, snacks, candies, etc. • Butter, margarine, shortening, coconut oil and other saturated and hydrogenated fats
Supplements	• 1 tsp. cod liver oil • 1000 μg methyl B_12_ • 1000 μg methylfolate • 5000 IU vitamin D_3_^a^ • 1 multivitamin/mineral tablet for men aged 50+ without iron	• 1 tsp. cod liver oil • 1000 μg methyl B_12_ • 1000 μg methylfolate • 5000 IU vitamin D_3_^a^ • 1 multivitamin/mineral tablet for men aged 50+ without iron

^a^Dose adjusted based on serum vitamin D

##### Study diet 1

The modified Paleolithic diet stresses more vegetables than other Paleolithic diets that have been studied previously, and it limits meat to 6–12 oz per day. Like other Paleolithic diets, it excludes all grain, legumes, and dairy (except for clarified butter or ghee) and additionally excludes eggs. Nightshade vegetables/spices will be excluded during the first 12 weeks on the diet and then reintroduced during the second 12 weeks on the diet, if the participant desires, to safely test tolerance to nightshades while under study team observation. Specific guidance will be provided to participants about how to reintroduce nightshades into the diet.

##### Study diet 2

The low saturated fat diet restricts saturated fat to ≤ 15 g per day and limits unsaturated fat to 20–50 g (4–10 teaspoons) per day. (This diet is promoted by the Swank MS Foundation [38] and includes more emphasis on vegetables and whole grains than the original Swank Diet.)

#### Dietary supplements

All study participants will follow the supplement regimen outlined in Table 1, with the exception of vitamin D_3_ (amount prescribed will vary on the basis of vitamin D level measured in the blood at visit 1 and subsequent visits as outlined in Table 2; target range, 40–80 ng/ml). After review of participant’s dietary supplement intake, study staff will ask participants to discontinue over-the-counter dietary supplements containing the study supplement ingredients (fish oil, vitamin D, vitamin B_12_, folate, and/or multivitamins) and use study supplements instead. Participants will be instructed to continue taking all other dietary supplements they have been using and not to begin any new supplements until after study completion. If the participant is asked to discontinue a supplement recommended by their physician or neurologist, the research team will send a letter to the treating physician, detailing which supplements have been discontinued and which supplements have been initiated as part of the study protocol.

**Table 2:** Vitamin D dosing based on blood level

If vitamin D > 100 ng/ml and elevated calcium (Ca^2+^) > 10.2 mg/dl • Call study participant and have them stop vitamin D immediately, increase water intake. a. If Ca^2+^ level was 10.2–10.5: call your local physician, or urgent care for guidance. b. If Ca^2+^ level was 10.6 or higher: go to the emergency room for evaluation and probable intravenous fluids. • Stop or do not start taking the cod liver oil or the multivitamin • When vitamin D < 80 ng/ml, resume vitamin D at a reduced dose and cod liver oil and multivitamin per local primary care or neurology guidance • Call and notify the study participant’s primary care/neurology team about the high vitamin D level • Send letter to participant and primary care/neurologist	
If vitamin D > 100 ng/ml and normal calcium Ca^2+^ 8.5–10.2 mg/dl • Study staff will call the participant and have them stop all vitamin D supplements. Participant will follow up with their primary care doctor or neurologist. • Stop or do not start taking cod liver oil but may continue multivitamin • When vitamin D < 80 ng/ml, may resume vitamin D at a reduced dose and cod liver oil and multivitamin per local primary care or neurology guidance • Call and notify the study participant’s primary care/neurology team about the high vitamin D level • Send letter to participant and primary care/neurologist	
If vitamin D between 81 and 100 ng/ml • Start or continue taking cod liver oil and multivitamin • Reduce total vitamin D intake by 50% ∘ The goal is half the dosage from all sources participant was taking at the time the blood measurement was made ∘ If the vitamin D_3_ supplement dosage was in fractions; then, round it down to the next available pill in the market. ∘ E.g., if the total taken is 7000; 1000 from MV, 1000 from calcium/vitamin D_3_ supplement and 5000 IU vitamin D_3_ we want to halve the dose; then the total amount to take becomes 3500. We will ask the participant to continue calcium/vitamin D_3_ (1000 IU), switch to our MV (1000 IU), start cod liver oil (400 IU), which totals 2400. The remaining vitamin D_3_ (3500–2400) is 1100 IU (round down to 1000 IU), so we prescribe a 1000-IU pill once daily. • Send letter to participant and primary care/neurologist	
If vitamin D 40 to 80 ng/ml At visits 2, 3, and 4: • Start or continue taking cod liver oil and multivitamin which contain about 1400 IU between the two supplements. Keep participant on current dose of vitamin D if in this range. At all visits: • Do not change vitamin D dose. E.g., if the participant is taking 4000 IU from their personal physician, participant should stay on that dose. If they were taking 5000 IU from their personal physician they should stay on that dose. If participant is taking more than 5000 IU or less than 4000 IU, participant should continue on that dose. • No need to start vitamin D_3_ supplement if not on any.	
If vitamin D value less than 40 ng/ml but more than 20 ng/ml If visit 1 blood result: • Wait until visit 2. At visit 2: • If not taking any vitamin D, begin 5000 IU daily. Participant will also begin or continue taking cod liver oil and multivitamin according to the supplement schedule. • If taking 4000 IU vitamin D_3_ or less daily, participant will be instructed to increase vitamin D_3_ to 5000 IU and add or continue taking cod liver and multivitamin according to the supplement schedule. • If the participant is already taking 5000 IU but does not take the supplement regularly (i.e., daily) they will be instructed to take the supplement every day and add or continue taking cod liver oil and multivitamin. • If taking 5000 IU or more they should add or continue taking cod liver oil and multivitamin. Discuss with Dr. Wahls for further guidance on the appropriate vitamin D dose.^a^ The patient and primary care/neurology team will be notified by letter of the lab results and the vitamin D, cod liver oil and multivitamin recommendations. • Send letter to participant and primary care/neurologist if participant is instructed to change vitamin D intake prescribed by medical personnel If visit 2 blood result: • Wait until visit 3 blood result. If visit 3 or 4 blood result: • And participant is taking 5000 IU vitamin D, cod liver oil and the multivitamin, discuss with Dr. Wahls for further guidance.^a^ An increase in the vitamin D dose will likely be recommended by Dr. Wahls and personalized results letters will be sent separately to the participant and their primary care/neurology team.	
If vitamin D value 20 ng/ ml or less If visit 1 or 2 blood result: • Refer participant back to primary care physician and neurologist, recommending the primary care physician or neurologist prescribe vitamin D_2_ 50,000 IU once per week until vitamin D level is greater than 40 ng/ml. Alternatively, the primary care physician may recommend 6000 to 10,000 IU vitamin D_3_ daily if preferred over vitamin D_2_. • Send letter to participant and primary care/neurologist • Call and notify participant that vitamin D level is severely low and advise them that a letter is being sent to them and their primary care/neurology team. *If visit 2, 3, or 4* • Call participant to clarify if the participant is taking any vitamin D supplements, cod liver oil or multivitamin and discuss with Dr. Wahls for further guidance. If already on vitamin D_3_ 5000 IU or a vitamin D_2_ supplement per their local medical team, the participant may continue the vitamin D until they see their primary care/neurology team for further guidance. Continue or add cod liver oil and multivitamin as scheduled. • Send letter to participant and primary care/neurologist	

^a^The total vitamin D_3_ from vitamin D_3_ supplement (including cod liver oil and the multivitamin) recommended by the study team should not exceed 7000 IU/d (cod liver oil and multivitamin have approximately 1400 IU daily combined) unless approved specifically by Dr. Wahls.

Abbreviation: *MV* Multivitamin

**Table 3:** Outcomes: change after 12-week intervention and 12 weeks of sustained intervention relative to observation period

Primary outcome measure:	
1. Perceived fatigue assessed by FSS.	
Secondary outcome measures:	
1. Perceived fatigue assessed by MFIS;	
2. Perceived fatigue assessed by FSMCF;	
3. Gait speed assessed by T25FW;	
4. Distance walked assessed by 6MWT;	
5. Speed of task completion in 9HPT;	
6. Number of correct responses assessed by SDMT-O;	
7. Quality of life as measured by MSQOL-54;	
8. Quality of life as measured by SF-36;	
9. MSISv2 score;	
10. Anxiety and depression as measured by HADS;	
11. Physical activity (average daily steps and average minutes spent sedentary, light intensity physical activity, moderate intensity physical activity and vigorous intensity physical activity) and sleep (total sleep time, sleep efficiency) as measured by the accelerometer;	
12. Sleep quality as measured by PSQI;	
13. Stress as measured by PSS-10;	
14. Physical activity as assessed by IPAQ-L;	
15. Cognitive performance as measured by PDQ;	
16. Change in mood as measured by the MHI.	
17. Nutritional adequacy of the diet as measured by food frequency questionnaire (FFQ) and food records;	
18. Changes in blood biomarkers (insulin, glucose, hemoglobin A1c, lipids including HDL, vitamin K, vitamin D, calcium, vitamin B_12_, folate, homocysteine, complete blood count with differential, Cardio IQ® [fatty acid levels]).	

*Abbreviations: 6MWT* 6-Minute walk test, *9HPT* 9-hole Pegboard Test, *Cardio IQ®* Omega-3 (EPA + DHA) Index, Omega-6/Omega-3 Ratio, EPA/Arachidonic Acid Ratio, Arachidonic Acid, EPA, and DHA, *DHA* Docosahexaenoic acid, *EPA* Eicosapentaenoic acid, *FFQ* Food Frequency Questionnaire, *FSMCF* Fatigue Scale for Motor and Cognitive Function, *FSS* Fatigue Severity Scale, *HADS* Hospital Anxiety and Depression Scale, *HDL* High Density Lipoprotein, *IPAQ-L* International Physical Activity Questionnaire–Long, *MFIS* Modified Fatigue Impact Scale, *MHI* Mental Health Inventory, *MSIS v2* Multiple Sclerosis Impact Scale version 2, *MSQ* Medical Symptoms Questionnaire, *MSQOL54* Multiple Sclerosis Quality of Life 54, *PDQ* Perceived Deficits Questionnaire, *PSQI* Pittsburgh Sleep Quality Index, *PSS 10* Perceived Stress Scale 10, *SDMT-O* Symbol Digit Modalities Test-Oral, *SF-36* 36-item Short Form Health Survey, *SPMSQ* Short Portable Mental Status Questionnaire, *T25FW* Timed 25-foot Walk

Study staff will provide participants with a list of supplements to purchase and will reimburse participants for all study supplements. Participants will be instructed to begin supplements on the 11th day of the diet to allow them time to purchase the supplements after returning home from visit 2. They will introduce one new supplement every third day to allow time to identify any potential adverse reactions. If side effects are noted, the participant will be told to stop the supplement and contact study staff.

#### Nutrition counseling

Following randomization, participants will receive two in-person and five phone nutrition counseling sessions with the intervention RD. Personalized emails with feedback will be sent after participants return the diet checklists every 4 weeks. Participants may contact the intervention RD at any time to receive additional support. A final in-person counseling session will be conducted at visit 4. Nutrition counseling will be based on the Self-Determination Theory and use the Motivational Interviewing (MI) communication approach [39–43]. The dietitians providing the intervention will be screened for MI aptitudes prior to being hired, and they will receive training in this area prior to study initiation. The dietitian will also receive ongoing consultative support throughout the study from an RD who is a member of the Motivational Interviewing Network of Trainers (MINT). A lesson plan was developed to guide the counseling session for each in-person study visit. Participants will be asked to consent to being audio recorded during in-person nutrition counseling sessions at visits 2 and 3. The visit 2 audio recordings will be reviewed by the MINT RD to verify fidelity to the study diet guidelines, which consist of explaining the recommended and not recommended foods for the intervention diet and rationale for key dietary components. The MINT RD will review all visit 2 audio recordings and 20% of visit 3 audio recordings using the OnePass instrument [44] to assess use of the MI approach. A mean OnePass score ≥ 4 indicates average competence in MI.

### Measures

The SPMSQ will be used at screening to assess organic brain deficit. Personal information will be collected at visit 1. A medication audit, health history, vital signs, and blood biomarkers (insulin, glucose, hemoglobin A1c, lipids including high-density lipoprotein, vitamin K, vitamin D, calcium, vitamin B_12_, folate, homocysteine, Cardio IQ® [Omega-3 (EPA + DHA) index, omega-6/omega-3 ratio, EPA/arachidonic acid ratio, arachidonic acid, EPA, and DHA]) will be collected at each visit. Table 3 summarizes the primary and secondary outcome measures.

*Perceived fatigue will be assessed with the following scales:*

1. The FSS [45]: a nine-item questionnaire measuring the severity of perceived fatigue and its effect on daily activities. Scores range from 0 to 7, with higher scores indicating greater perceived fatigue severity. Obtaining scores every 4 weeks will provide more assessment points for the primary outcome measure and will improve the statistical power for hypothesis testing.
2. Modified Fatigue Impact Scale (MFIS) [46], a 21-item assessment of the effects of fatigue related to physical, cognitive, and psychosocial functioning.
3. Fatigue Scale for Motor and Cognitive Functions (FSMCF), a 20-item measure of cognitive and motor fatigue for people with MS.

*Motor function (gait and hand) will be assessed with the following tests:*

1. T25FW, a timed 25-ft walk to quantify mobility and leg function
2. 6MWT assesses distance walked in 6 minutes as a submaximal test of aerobic capacity.
3. 9HPT is a quantitative test of upper extremity function (hand) in which patients are timed as they put nine pegs into holes in a block and then remove them one at a time. The test is conducted twice with the nondominant hand and twice with the dominant hand.

*Cognitive function will be assessed by:*

1. SDMT-O is a screening tool for organic cerebral dysfunction.
2. Perceived Deficits Questionnaire (PDQ) is a self-report measure of cognitive dysfunction specifically for MS.

*QOL will be assessed by:*

1. MSQOL54 [47] is a multidimensional health-related QOL measure that includes both generic and MS-specific items.
2. SF-36 is a generic measure of health-related QOL.
3. HADS [48] is an instrument used to assess mood and detect states of depression and anxiety.
4. Mental Health Inventory (MHI) [49] provides an assessment of anxiety, depression, behavioral control, positive affect, and general distress.
5. Multiple Sclerosis Impact Scale version 2 (MSIS v2) is a 29-item self-report measure of the impact of MS on day-to-day activities, including both physical and psychosocial aspects.
6. Perceived Stress Scale 10 (PSS-10) [50] measures the degree to which situations in one’s life are perceived as stressful.

*Physical activity and sleep will be assessed by:*

1. Accelerometers (GT9X Link; ActiGraph, Pensacola, FL, USA) will be worn on the nondominant wrist during the 7-day food-recording period (including sleep time). Outcomes measured by accelerometer will include daily steps, time spent in light-intensity activity, time spent in moderate-intensity activity, time spent sedentary, sleep time, and sleep efficiency.
2. International Physical Activity Questionnaire Long, (IPAQ-L) [51], a physical activity assessment.
3. Pittsburgh Sleep Quality Index (PSQI) [52], a 19-item self-report sleep quality assessment.

*Diet quality will be assessed by:*

1. Three-day weighed food records to assess nutrient intake. Nutrient intake will be calculated using the most current version of the Nutrition Data System for Research software (Nutrition Coordinating Center, University of Minnesota, Minneapolis, MN, USA). (*Note*: Initially, 7-day records were collected. Based on participant feedback, and confirmed with the Minnesota Nutrition Coordinating Center, consecutive 3-day records are in used in a standard manner to assess nutrient intake [53–55].)
2. Harvard Food Frequency Questionnaire (FFQ) is used to obtain information regarding the frequency and portion size of food and beverages consumed over the past year (visit 1) and the past 12 weeks (visits 2–4).

*Social support for eating will be assessed with:*

1. A version of Social Support for Eating Habits Scale [56], modified to include factors that are key to adherence by family members and friends, will assess social support for eating. These factors were identified in qualitative interviews with participants from our multimodal pilot study [4]. They include food purchasing, meal preparation, snack availability, and modeling study diet adherence while in the presence of the study participant.

To further monitor the health of participants, the following data variables will be collected:

- Health Practices Survey, a subset of questions asking about use of complementary therapies, including massage, meditation, yoga, and Tai Chi derived from the complementary health approaches section of National Health Interview Survey [57].
- Side effects questionnaire to assess new or worsening symptoms that might be attributed to diet or supplement changes (*see* Additional file 2: Appendix 1).
- “Your Eating Experience” questionnaire, developed by our research team to collect the following information: how often foods that are to be avoided were consumed during the past 4 weeks, what made it difficult to avoid those foods, and the factors that helped the participant follow the dietary guidelines (*see* Additional file 3: Appendix 2).

### Assessments and visit schedule

#### Visit 1 – week 1

Participants will fast for 12 hours prior to visit 1. Eligibility criteria will be checked, and the participant will be consented (Additional file 4: Appendix 3). Height and weight will be measured to confirm a BMI ≥ 19. If participants qualify for the study, their vital signs will be measured, and fasting blood will be drawn. Questionnaires including a medication audit and motor and cognitive testing will be completed, as listed in Fig. 2, and personal information such as duration of MS and handedness will be recorded. Participants will be instructed on how to (1) keep a 3-day food record and use a loaned food scale for weighing the amount of food eaten, (2) complete an FFQ, and (3) wear an accelerometer.

Participants will complete questionnaires, as listed in Fig. 2, via a secure Research Electronic Data Capture (REDCap) system at home 1–3 days after the study visit. Participants will wear an accelerometer for 7 continuous days (24 h/d) following this visit and record the foods, beverages, and dietary supplements consumed between Thursday and Saturday following visit 1. The accelerometer and dietary forms will be returned by mail.

#### Visit 2 – week 12

Participants will wear an accelerometer for the 7 days prior to visit 2 and will record the foods, beverages, and dietary supplements consumed between Thursday and Saturday prior to the visit. Participants will complete questionnaires (as outlined in Fig. 2) 1–3 days prior to visit 2 using REDCap. Participants will fast for 12 hours prior to visit 2. Vital signs, blood, motor and cognitive tests, and questionnaires will be completed at visit 2 (*see* Fig. 2). Food records, FFQs, and accelerometer data, will be reviewed for completeness. Participants will be randomized to one of the study diets.

The study participant and their family member(s) or adult companion will meet with an RD who will provide individualized nutrition counseling on the participant’s assigned study diet. The family/adult companion will be advised that, during our previous studies, we have observed that the study participants who have had their families adopt the study diet along with the study participants have had greater levels of success with adopting and sustaining the intervention diets. The family/adult companion will be advised that they are expected to be supportive of eating study-compliant foods.

Participants will view a brief video (< 20 min) that provides background information about the study diet to which they were randomized. Participants will receive an intervention food guide developed by study staff that describes their study diet, as well as *The Multiple Sclerosis Diet Book* [58] (low saturated fat diet) or *The Wahls Protocol* [59] (modified Paleolithic diet), both of which contain sample menus and recipes to assist with meal planning. Additional materials, such as grocery lists, will be provided to assist with diet adoption. A diet checklist that offers guidance to the participant in the form of prompts to eat the foods that are recommended and to avoid those that are not recommended will be provided. The participants and their adult companions will be given a study-adherent meal and practice recording the meal in the diet checklist to facilitate the learning of the principles of the assigned study diet and how to use the daily diet checklist.

Participants will be given a set of diet checklists to complete daily and a 3-day weighed food record booklet as well as an FFQ to complete prior to their next study visit. A self-addressed, stamped envelope will be provided so that participants can return their diet checklists at weeks 16 and 20 for the study dietitian to review. Upon receiving the week 20 checklist, the RD will send an email to acknowledge receipt and provide assessment comments. Participants will be asked to continue their usual physical activity, medication, and stress reduction regimens during the next 24 weeks unless their physician or physical therapist recommends changes.

The study RD will continue to coach the participant via coaching calls 2–3 days after visit 2 and then weekly for 3 weeks. A final coaching call will be made after receipt of the first 4-week diet checklist booklet.

#### Visit 3 – week 24

Participants will wear an accelerometer for the 7 days prior to visit 3 and will record the foods, beverages, and dietary supplements consumed between Thursday and Saturday prior to the visit. Participants will again complete questionnaires (as outlined in Fig. 2) 1–3 days prior to visit 3 using REDCap [60]. Participants will fast for 12 hours prior to visit 3. Vital signs, blood, motor and cognitive tests, and questionnaires will be completed at visit 3 (*see* Fig. 2). Food records, FFQs, and accelerometer data will be reviewed for completeness. The participant will meet with the study dietitian, who will review the diet checklists and answer questions. For participants randomized to the modified Paleolithic diet, the RD will encourage but not require the participant to reintroduce nightshades to the diet. If the participant elects to reintroduce nightshades, one nightshade food will be introduced on 1 day using this process: consume one bite of the food and wait 3 hours; consume ½ cup and wait 3 hours; consume 1 cup of food and wait 6 days. Symptoms will be recorded for a total of 7 days. If no symptoms have appeared by the end of 7 days, the food may be added back to the diet, and another food can be tested.

Participants will be given a fresh set of diet checklists to complete daily and a 3-day weighed food record booklet and FFQ to complete prior to their next study visit. A self-addressed, stamped envelope will be provided so that participants can return their diet checklists at weeks 28 and 32 for the study RD to review. An email will be sent to acknowledge receipt and provide assessment comments. The RD will not make coaching calls during this time, but study participants may contact the RD to request support. Participants will be reminded to continue their usual physical activity, medication, supplement, and stress reduction regimens during the next 12 weeks unless their physician or physical therapist directs them to make changes.

#### Visit 4 – week 36

Participants will wear an accelerometer for the 7 days prior to visit 4 and will record the foods, beverages, and dietary supplements consumed between Thursday and Saturday prior to the visit. Participants will again complete some questionnaires (as outlined in Fig. 2) 1–3 days prior to visit 4 using REDCap. Participants will fast for 12 hours prior to visit 4. Vital signs, blood, motor and cognitive tests, and questionnaires will be completed at visit 4 (*see* Fig. 2). Food records, FFQs, and accelerometer data will be reviewed for completeness. Participants will view two videos from the principal investigator: a thank-you video and an educational video about environmental factors that contribute to health. The participant will then meet with the study RD, who will review the diet checklists and answer questions.

### Risks and safety monitoring during intervention

#### Weight monitoring

If a participant’s BMI at visit 1 is < 19 kg/m^2^, they are ineligible for the study. If BMI at visit 2 or 3 is < 18.5 kg/m^2^, the participants will be asked to weigh themselves at home 1 and 2 weeks after the study visit (in the morning after their first void wearing street clothing) and report that information to the study coordinator/study team. If the BMI remains < 18.5 kg/m^2^ for 2 consecutive weeks:

1. The intervention dietitian will conduct a special telephone coaching call to discuss strategies for increasing their calorie intake. The dietitian will make a second telephone call 1 week after the first call to provide continued support. Additional calls will be based on participant need and dietitian assessment. The participant will be instructed to continue weighing themselves weekly and reporting that information to the study team.
2. The participant will be advised to follow up with their personal physician regarding the weight loss.

If the BMI increases to 19 kg/m^2^, the weekly weight monitoring will be discontinued. If a participant’s BMI at study visit 2 is 18.5–19 kg/m^2^, the participant may be at risk for additional weight loss, so the dietitian will alert them of this possibility and briefly discuss strategies to prevent weight loss.

The study team will report to the Data and Safety Monitoring Board (DSMB) the number of participants currently being monitored for low BMI and the corrective actions taken by study staff. The DSMB will review the BMIs of all participants quarterly along with any corrective action taken by study staff. The DSMB is constituted and managed by the NMSS and will review the adverse events, significant adverse events, and laboratory values greater than 10% outside the reference range. In addition to the quarterly reports provided to the DSMB, trial auditing procedures will also include a closed report from the study statistician with more details related to diet assignment and adverse events. The data management application also provides data manipulation and user activity audit trails. A medical monitoring board composed of two UI faculty physicians will receive all adverse events sheets. A data monitoring committee has not been constituted. Guidelines for stopping participation due to adverse events, patient decision to withdraw, or treatment medical provider recommendation have been established. Coaching and contact with study dietitian and motivational interviewing. Subjects who elect or are obligated to discontinue the study diet will be asked to complete the study forms and the motor assessments through 36 weeks.

### Data management

Study data will be collected and managed using REDCap [60] tools hosted at UI. REDCap is a secure, web-based application designed to support data capture for research studies, and it provides (1) an intuitive interface for validated data entry, (2) audit trails for tracking data manipulation and export procedures, (3) automated export procedures for seamless data downloads to common statistical packages, and (4) procedures for importing data from external sources. Study participants will log into REDCap using a secure web-based portal to answer study questionnaires from home. Study staff will use REDCap for manual entry of laboratory and other data and interviewer-administered questionnaires.

### Power and sample size justification

Table 4 displays the mean minimum clinically important difference that can be detected for primary and secondary outcomes to be assessed in the study. The table also includes the SD and correlation between observations over time, with 34 patients in each group after attrition, and an alpha value of 0.05. The minimum differences and SDs are derived from the preliminary studies and peer-reviewed published MS longitudinal studies. Forty-five patients will be recruited in each group, assuming a 25% attrition rate, to attain the 34 patients needed in each group.

**Table 4:** Power and sample size justification

Variable	Within Study Groups			Between Study Groups at Last Visit		
	MMCD	SD	Correlation	MMCD	SD Group A	SD Group B
Power is ≥0.85 for the primary outcome						
FSS-9	1.2–2.0	1.1–1.4	0.6	0.7 [35]	0.9	1.1
Power is ≥0.90 for secondary outcomes						
MSQOL-54						
PHC	11.5	16.0	0.6	17.0	17.0	19.0
MHC	12.0	17.0	0.5	18.0	18.0	19.0
6 min walk test	35	50.0	0.5	45.0	45.0	50.0
Timed 25 ft walk	2.9	4.0	0.5	4.0	4.0	4.3
9 hole peg test	5.5	8.0	0.5	8.0	8.1	8.3
PASAT^a^	4.2	6.0	0.5	5.8	6.0	6.3

*Abbreviations: FSS* Fatigue Severity Scale, *MSQOL-54* Multiple Sclerosis Quality of Life 54, *PHC* MSQOL-54 physical health composite score, *MHC* MSQOL-54 mentalhealth composite score, *PASAT* Paced Auditory Serial Addition Test, *MMCD* Minimun Mean Clinical Difference

^a^Power and sample size calculations based on PASAT. Per NMSS, SDMT-O replaces the PASAT measure in this study

### Data analysis

All analyses will be completed by a masked statistician who will not have access to study diet assignment. Data analysis will include both intention-to-treat and per-protocol analytic methods. Data for this study will be analyzed using SAS® 9.4 software (SAS Institute, Cary, NC, USA). Statistical significance will be set at α ≤ 0.05. Descriptive statistics will be calculated for all variables at all data collection times. Outliers will be checked for accuracy and possible entry errors. Respondents who drop out of the study will be compared with those who complete the study so that differences in baseline characteristics that may affect the study results will be detected. Patterns of missing data (missing at random, not missing at random) will be investigated [61]. The analytic methods will account for the type of missing data. The distributions of continuous variables will be evaluated for normality and homogeneity of variance. Appropriate transformations will be applied as needed, and/or statistical analyses for nonnormal data will be performed. Collinearity among fixed effect variables will be examined.

Repeated-measures analyses will use generalized linear mixed models methods to test for within-group and between-group changes in both primary and secondary outcomes. Intraclass correlations for repeated measures within patients will be included in the model structure for analyses. Mixed models for both continuous and categorical outcomes will have both random and fixed effects. Fixed effects will include characteristics such age, sex, intervention group, time indicators, and group-by-time interactions. Intercepts and slopes may be considered as random effects.

## Discussion

Clinical studies of diet in MS are needed because there is uncertainty about the optimal dietary approach to reducing MS-related fatigue. Randomized trials that include assignment to a control or standard care group are the preferred design; however, previous studies including dietary approaches to reduce MS-related symptoms suggest increased participant adherence when a study diet is offered to all participants because dropout is high among nondiet control group participants. Crossover study designs have also been used to test dietary hypotheses. However, we have observed MS symptom reductions among participants following a modified Paleolithic diet and marked increases in MS-related symptoms among those who returned to eating gluten-containing grains or casein-containing dairy products. Therefore, to most effectively and ethically study such dietary approaches, the comparison of two study diets is a better approach than a crossover design for this population.

Maintaining differences between the dietary groups may be challenging because participants may want to modify their eating patterns to make them similar to the diet to which they are not assigned. That is, participants in the low saturated fat diet group may wish to eliminate gluten and dairy, and those in the modified Paleolithic diet group may elect to greatly restrict saturated fat intake. To minimize this, the dietary intervention guide for the low saturated fat diet encourages consumption of gluten-containing grains, and the modified Paleolithic diet encourages consumption of foods containing saturated fat. Additionally, physical symptoms related to consumption of gluten or a reluctance to eat saturated fat are included in the inclusion/exclusion criteria.

Both study groups are likely to experience reductions in MS-related symptoms. Differences within and between groups will be assessed for clinical significance, supported by statistical analyses. Because the primary outcome measure is a self-reported measure and not an objectively measured laboratory or imaging result, the NMSS requested two modifications in the study design. First, a 12-week observation period was added, during which participants continue to eat their usual diet, so that each participant can be her or his own control in the repeated-measures analyses. Second, an accelerometer measure was added to the study to provide an objective assessment of the change in physical activity, sleep duration, and sleep quality.

Given the anecdotal reports regarding improvement in MS symptoms with dietary changes, results from this study will contribute to understanding of the optimal dietary approach for reducing MS-related fatigue. Findings of the study as well as any challenges observed will be submitted to journals for publication. Published papers will be listed on the Wahls research lab website (https://wahls.lab.uiowa.edu/publications).

### Trial status

Recruitment commenced August 24, 2016. We have consented 58 participants to date.

## Additional files

Additional file 1: SPIRIT Checklist. (DOC 121 kb)

Additional file 2: Appendix 1. Side Effects Survey. (PDF 47 kb)

Additional file 3: Appendix 2. Your Eating Experience Questionnaire. (PDF 56 kb)

Additional file 4: Appendix 3. Study Consent. (PDF 259 kb)

## Abbreviations

6MWT: 6-Minute walk test
9HPT: 9-Hole Pegboard Test
ADA: American Diabetes Association
BMI: Body mass index
Cardio IQ®: Omega-3 (EPA + DHA) index, omega-6/omega-3 ratio, EPA/arachidonic acid ratio, arachidonic acid, EPA, and DHA
DHA: Docosahexaenoic acid
DSMB: Data and Safety Monitoring Board
EPA: Eicosapentaenoic acid
FFQ: Food Frequency Questionnaire
FSMCF: Fatigue Scale for Motor and Cognitive Function
FSS: Fatigue Severity Scale
HADS: Hospital Anxiety and Depression Scale
HDL: High-density lipoprotein
IPAQ-L: International Physical Activity Questionnaire–Long
IRB: Institutional review board
MFIS: Modified Fatigue Impact Scale
MHI: Mental Health Inventory
MI: Motivational Interviewing
MINT: Motivational Interviewing Network of Trainers
MRI: Magnetic resonance imaging
MS: Multiple sclerosis
MSIS v2: Multiple Sclerosis Impact Scale version 2
MSQ: Medical Symptoms Questionnaire
MSQOL54: Multiple Sclerosis Quality of Life 54
NMSS: National Multiple Sclerosis Society
PDQ: Perceived Deficits Questionnaire
PSQI: Pittsburgh Sleep Quality Index
PSS 10: Perceived Stress Scale 10
QOL: Quality of life
RD: Registered dietitian
REDCap: Research Electronic Data Capture
RRMS: Relapsing-remitting multiple sclerosis
SDMT-O: Symbol Digit Modalities Test–Oral
SF36: 36-Item Short Form Health Survey
SPMSQ: Short Portable Mental Status Questionnaire
T25FW: Timed 25-foot walk
UI: University of Iowa
