# Supplementary material for: Dietary approaches to treat MS-related fatigue: comparing the modified Paleolithic (Wahls Elimination) and low saturated fat (Swank) diets on perceived fatigue in persons with relapsing-remitting multiple sclerosis: study protocol for a randomized controlled trial
Source: Trials. 2018 Jun 4;19:309. doi: 10.1186/s13063-018-2680-x (PMC5987638; doi:10.1186/s13063-018-2680-x)
Supplement: Supplementary file 2 — Appendix 1. Side Effects Survey. (PDF 47 kb) [file 13063_2018_2680_MOESM2_ESM.pdf]

Side Effects Survey

Please complete the survey below.

Thank you!

Please fill out this survey even if you have not begun the study diet.

Date form completed ________________________________

Please indicate if you have experienced any of these symptoms since you began the study diet and study dietary supplements and rate the severity of the symptoms. These could be new symptoms that began after you started the diet and supplements OR symptoms that were present before you started the diet but that have WORSENED after you started the study diet .

If you experienced a symptom but you do not attribute it to the dietary changes you made, mark None.

	None	Mild	Moderate	Severe
Body aches	◯	◯	◯	◯
Joint pain	◯	◯	◯	◯
Abdominal pain	◯	◯	◯	◯
Chest pain	◯	◯	◯	◯
Palpitations	◯	◯	◯	◯
Heartburn	◯	◯	◯	◯
Bruising	◯	◯	◯	◯
Easy bleeding	◯	◯	◯	◯
Bloating	◯	◯	◯	◯
Belching	◯	◯	◯	◯
Vomiting	◯	◯	◯	◯
Flatulence (gas)	◯	◯	◯	◯
Diarrhea	◯	◯	◯	◯
Constipation	◯	◯	◯	◯
Headache	◯	◯	◯	◯
Nausea	◯	◯	◯	◯
Dry skin/hair	◯	◯	◯	◯
Skin rash	◯	◯	◯	◯
Dry mouth	◯	◯	◯	◯
Bad breath	◯	◯	◯	◯
Bad taste in mouth	◯	◯	◯	◯

Worsening of mood	○	○	○	○

Other Symptom not listed above #1: Describe the symptom
(Hit "Tab" button when done)

____

Please Rate the Intensity of Other Symptom #1

○ None
○ Mild
○ Moderate
○ Severe

Other Symptom not listed above #2: Describe the symptom
(Hit "Tab" button when done)

____

Please Rate the Intensity of Other Symptom #2

○ None
○ Mild
○ Moderate
○ Severe

Other Symptom not listed above #3: Describe the symptom
(Hit "Tab" button when done)

____

Please Rate the Intensity of Other Symptom #3

○ None
○ Mild
○ Moderate
○ Severe

Other Symptom not listed above #4: Describe the symptom
(Hit "Tab" button when done)

____

Please Rate the Intensity of Other Symptom #4

○ None
○ Mild
○ Moderate
○ Severe

Other Symptom not listed above #5: Describe the symptom
(Hit "Tab" button when done)

____

Please Rate the Intensity of Other Symptom #5

○ None
○ Mild
○ Moderate
○ Severe

Please provide any comments about the side effects you attribute to the diet and/or dietary supplements.

Comments:

____
