# Supplementary material for: Dietary approaches to treat MS-related fatigue: comparing the modified Paleolithic (Wahls Elimination) and low saturated fat (Swank) diets on perceived fatigue in persons with relapsing-remitting multiple sclerosis: study protocol for a randomized controlled trial
Source: Trials. 2018 Jun 4;19:309. doi: 10.1186/s13063-018-2680-x (PMC5987638; doi:10.1186/s13063-018-2680-x)
Supplement: Supplementary file 3 — Appendix 2. Your Eating Experience Questionnaire. (PDF 56 kb) [file 13063_2018_2680_MOESM3_ESM.pdf]

Your Eating Experiences Questionnaire 2

Please complete the survey below.

Thank you!

Date You Completed this Questionnaire ________________________________

Please select your study visit

- ◯ Visit 1
- ◯ Visit 2
- ◯ Visit 3
- ◯ Visit 4
- ◯ long-term follow up study

On average, how often during the past 4 weeks did you eat the following foods?

1. Grains

Grains include bread, rolls, bagels, pancakes, rice, pasta, tortillas, hot and cold cereal, crackers, etc.

One serving =
1/2 cup rice or pasta
1 slice bread
1/2 hamburger bun
1 cup or 1 oz cold cereal
1/2 cup cooked hot cereal

- ◯ Never or less than once per month
- ◯ 1 per month
- ◯ 2-3 per month
- ◯ 1 per week
- ◯ 2 per week
- ◯ 3-4 per week
- ◯ 5-6 per week
- ◯ 1 per day
- ◯ 2+ per day

What grains did you eat most often?

____

2. Vegetables

one serving =
2 cups RAW LEAFY greens
1 cup other raw or cooked vegetables

- ◯ Never or less than once per month
- ◯ 1 per month
- ◯ 2-3 per month
- ◯ 1 per week
- ◯ 2 per week
- ◯ 3-4 per week
- ◯ 5-6 per week
- ◯ 1 per day
- ◯ 2 per day
- ◯ 3-5 per day
- ◯ 6-9 per day
- ◯ more than 9 per day

What vegetables did you eat most often?

____

3. Red meat (beef, pork, lamb), dark meat poultry, and processed meat

one serving =
4 oz cooked meat

- ◯ Never or less than once per month
- ◯ 1 per month
- ◯ 2-3 per month
- ◯ 1 per week
- ◯ 2 per week
- ◯ 3-4 per week
- ◯ 5-6 per week
- ◯ 1 per day
- ◯ 2+ per day

What type of red meat, dark meat poultry or processed meat did you eat most often?

____

4. Coconut oil or coconut milk, bacon fat, or ghee

one serving =
1 tablespoon fat
1/3 cup coconut milk (full fat)

- ◯ Never or less than once per month
- ◯ 1 per month
- ◯ 2-3 per month
- ◯ 1 per week
- ◯ 2 per week
- ◯ 3-4 per week
- ◯ 5-6 per week
- ◯ 1 per day
- ◯ 2+ per day

Which of these did you use most often (coconut oil, full fat coconut milk, bacon fat or ghee)?

____

5. Egg Yolks

one serving =
1 egg yolk

- ◯ Never or less than once per month
- ◯ 1 per month
- ◯ 2-3 per month
- ◯ 1 per week
- ◯ 2 per week
- ◯ 3-4 per week
- ◯ 5-6 per week
- ◯ 1 per day
- ◯ 2+ per day

6. Skim or Fat-Free Dairy Product made from cow, goat or mare's milk

one serving =
1 cup skim milk or fat-free yogurt
1.5 oz fat-free natural cheese
2 oz fat-free processed cheese
1/2 cup fat-free ice cream

- ◯ Never or less than once per month
- ◯ 1 per month
- ◯ 2-3 per month
- ◯ 1 per week
- ◯ 2 per week
- ◯ 3-4 per week
- ◯ 5-6 per week
- ◯ 1 per day
- ◯ 2+ per day

7. Higher Fat Dairy Products made from cow, goat or mare's milk

one serving =
1 cup 1%, 2% or whole milk or yogurt
1.5 oz natural cheese
2 oz processed cheese
1/2 cup ice cream

- ◯ Never or less than once per month
- ◯ 1 per month
- ◯ 2-3 per month
- ◯ 1 per week
- ◯ 2 per week
- ◯ 3-4 per week
- ◯ 5-6 per week
- ◯ 1 per day
- ◯ 2+ per day

8. Legumes (Beans, Peas, Soy, Lentils, Peanuts)

one serving =
1/2 cup cooked beans, peas, lentils, tofu
1 cup soy milk or soy yogurt
1/2 oz peanuts
1 tablespoon peanut butter

- ◯ Never or less than once per month
- ◯ 1 per month
- ◯ 2-3 per month
- ◯ 1 per week
- ◯ 2 per week
- ◯ 3-4 per week
- ◯ 5-6 per week
- ◯ 1 per day
- ◯ 2+ per day

9. Gluten-Free Grains

This includes rice, oats, quinoa, buckwheat, amaranth

one serving =
1/2 cup cooked rice, gluten-free grain or gluten-free pasta
1 slice gluten-free bread
1/2 gluten-free hamburger bun
1 cup or 1 oz gluten-free cold cereal

- ◯ Never or less than once per month
- ◯ 1 per month
- ◯ 2-3 per month
- ◯ 1 per week
- ◯ 2 per week
- ◯ 3-4 per week
- ◯ 5-6 per week
- ◯ 1 per day
- ◯ 2+ per day

10. Grains with Gluten

This includes foods made with wheat, rye, and barley like breads, pasta, pancakes, and cereals.

one serving =
1/2 cup cooked pasta or barley
1 slice bread
1/2 hamburger bun
1 cup or 1 oz cold cereal made from wheat, rye or barley

- ◯ Never or less than once per month
- ◯ 1 per month
- ◯ 2-3 per month
- ◯ 1 per week
- ◯ 2 per week
- ◯ 3-4 per week
- ◯ 5-6 per week
- ◯ 1 per day
- ◯ 2+ per day

11. Foods and beverages containing artificial sweeteners, sugar, honey, maple syrup, high fructose corn syrup, and other sweetening agents or the addition of these sweeteners to your food

one serving =
1 cup beverage
1 serving cake, cookies, pies, candy
1 tsp sugar, artificial sweetener
1 tsp honey, jam, molasses

- ◯ Never or less than once per month
- ◯ 1 per month
- ◯ 2-3 per month
- ◯ 1 per week
- ◯ 2 per week
- ◯ 3-4 per week
- ◯ 5-6 per week
- ◯ 1 per day
- ◯ 2+ per day

12. Commercial desserts, snack crackers and chips, chocolate candy, pizza

one serving =
1 oz cookie
1 oz snack crackers
1.4 oz chocolate candy
1 piece pie or cake
1 slice pizza

- ◯ Never or less than once per month
- ◯ 1 per month
- ◯ 2-3 per month
- ◯ 1 per week
- ◯ 2 per week
- ◯ 3-4 per week
- ◯ 5-6 per week
- ◯ 1 per day
- ◯ 2+ per day

13. Butter and Margarine added to foods

one serving =
1 tbsp butter or margarine

- ◯ Never or less than once per month
- ◯ 1 per month
- ◯ 2-3 per month
- ◯ 1 per week
- ◯ 2 per week
- ◯ 3-4 per week
- ◯ 5-6 per week
- ◯ 1 per day
- ◯ 2+ per day

14. Nightshade Vegetables/Spices

This includes tomatoes, white potatoes, eggplant, sweet peppers (any color), hot peppers, paprika, cayenne pepper, chili powder

one serving =
1 cup cooked or raw vegetable
1/8 tsp paprika, cayenne pepper, chili powder, etc.

- ◯ Never or less than once per month
- ◯ 1 per month
- ◯ 2-3 per month
- ◯ 1 per week
- ◯ 2 per week
- ◯ 3-4 per week
- ◯ 5-6 per week
- ◯ 1 per day
- ◯ 2+ per day

Thinking back over the past 4 weeks, what made it difficult to follow your study diet?

____

Thinking back over the past 4 weeks, what helped you to follow your study diet?

____

What eating pattern do you think is best for you?

____

Thinking about the eating pattern you think is best for you, what made it difficult to eat these foods?

____

Thinking about the eating pattern you think is best for you, what helped you to eat these foods?

____

Other Comments

____
