# Supplementary material for: Dietary approaches to treat MS-related fatigue: comparing the modified Paleolithic (Wahls Elimination) and low saturated fat (Swank) diets on perceived fatigue in persons with relapsing-remitting multiple sclerosis: study protocol for a randomized controlled trial
Source: Trials. 2018 Jun 4;19:309. doi: 10.1186/s13063-018-2680-x (PMC5987638; doi:10.1186/s13063-018-2680-x)
Supplement: Supplementary file 4 — Appendix 3. Study Consent. (PDF 259 kb) [file 13063_2018_2680_MOESM4_ESM.pdf]

INFORMED CONSENT DOCUMENT

Project Title: **Dietary Approaches to Treat Multiple Sclerosis Related Fatigue (Waves Study)**

Principal Investigator: Terry Wahls MD
Co-Principal Investigator: Linda Snetselaar PhD, RD, LD

Research Team Contact: Mary Ehlinger, Clinical Research Coordinator can be reached at 319-384-5002.

This consent form describes the research study to help you decide if you want to participate. This form provides important information about what you will be asked to do during the study, about the risks and benefits of the study, and about your rights as a research participant.

If you have any questions about or do not understand something in this form, you should ask the research team for more information.

You should discuss your participation with anyone you choose such as family or friends.

Do not agree to participate in this study unless the research team has answered your questions and you decide that you want to be part of this study.

WHAT IS THE PURPOSE OF THIS STUDY?

This is a research study. We are inviting you to participate in this research study because you have relapsing remitting multiple sclerosis (MS) and multiple sclerosis related fatigue and have expressed interest in a dietary approach to treating your disease. After we review this informed consent document with you and answer any questions you have, we will assess your understanding of the study by asking you some questions. If you answer these questions to our satisfaction and you wish to participate in this study, we will invite you to sign this informed consent document to signify your desire to participate in the study.

This study consists of two study phases: Usual diet phase and Intervention diet phase. The purpose of the **Usual diet phase** is to 1) determine if you are capable of completing detailed weighed food records which are required to be eligible to participate in the Intervention diet phase and 2) collect information about your usual dietary habits and fatigue level. The purpose of the **Intervention diet phase** is to compare two dietary approaches to reduce multiple sclerosis related fatigue.

One diet is a low saturated fat diet that eliminates beef and pork and includes whole grains, fat free dairy, vegetables and fruits (Swank) and the other is a modified paleo diet (Wahls Elimination) that eliminates all grains, legumes (e.g., beans), eggs, dairy, and nightshade vegetables (tomatoes, potatoes, eggplant, peppers) and stresses more vegetables and meat in the diet. Our hypothesis is that consuming the study diets will reduce multiple sclerosis related fatigue, improve energy and improve quality of life. Participants will be randomized 1:1; this means that you will have an equal chance of being assigned to either study diet; **therefore, we need participants who are willing to follow either diet and make changes in the foods they eat.**

HOW MANY PEOPLE WILL PARTICIPATE?

Approximately 1000 people with multiple sclerosis will take part in this study conducted by

investigators at the University of Iowa. Participants could be a part of a research registry, be asked for permission to contact their neurologist and/or be enrolled in this study.

HOW LONG WILL I BE IN THIS STUDY?

If you agree to participate in the study your involvement will last approximately 36 weeks with 4 visits to the Preventive Intervention Center (PIC) at the University of Iowa Hospitals and Clinics every 12 weeks. Visits will last 4 to 6 hours, depending on the visit. The Usual diet phase of the study (visits 1 and 2) lasts for 12 weeks. If you are eligible to continue into the Intervention Diet phase, your involvement will continue for 24 weeks with two additional visits at 24 and 36 weeks (visits 3 and 4).

WHAT WILL HAPPEN DURING THIS STUDY?

During this study you will continue to take any medications prescribed by your doctor and follow your usual exercise and self-care routine. **We ask that you not start any new dietary supplements, exercise or stress reduction programs unless directed to by your physician or physical therapist.**

If you experience any adverse events during the study, we will ask for information regarding the event in order to fill out our adverse events form for documentation.

Blood collection. To participate in this study you must agree to have your blood drawn to monitor potential changes in your nutritional biomarkers (blood tests of your nutritional status). Any changes in the biomarkers will be followed and compared to the presence or absence of changes in your physical condition, thinking ability and mood over time. When you arrive at your study visit, we will have you start drinking water. This will make it easier for staff to draw blood. Approximately 7 teaspoons of blood will be drawn at each visit. This blood draw, along with the gathering of other vitals (heart rate, blood pressure, etc.), will take 15-25 mins to complete. Your blood samples will be labeled with your study ID or a random number and transported immediately to a laboratory in the VA hospital for analysis or frozen and stored in the Pharmacy Building or College of Nursing for future analysis. The future analysis of your blood samples may be done at the University of Iowa or the University of Nebraska Medical Center by IRB approved study team members. **Blood is a very important component to this study. If we are unable to draw blood at a visit, or if the blood was not properly processed by lab staff, we will ask you to have blood drawn at a medical facility in your home town and sent to our lab. The study team will communicate with your medical facility to coordinate what bloods to draw, payment and transportation. If you do not feel comfortable having blood drawn or are unable to have blood drawn during the Usual diet phase, the study team can end your participation in the study and you will not move on to the Intervention diet phase.**

Physical Motor Tasks. You will complete the MS Functional composite. This test consists of a 25 foot walk test which is completed twice and a test of hand coordination that is completed with both the right and left hands. You will also complete a symbol digit task that asks you to substitute a number for meaningless geometric shapes based on a key that assigns a specific number to each shape. You can rest after the tests. Then you will complete the 6 minute walk test. You will rest after the test. These tests will be conducted at each visit. In order to prevent your fatigue level from rising before the motor tests, we will transport you in a wheelchair to the various parts of the visit that require us to leave the study office. This includes going to the blood draw room and to the hallway where we will perform the 6

minute walk test. **Please do not tell the study staff conducting these measurements anything about the study diet to which you are assigned; the staff conducting these tests must not know which diet you are following.**

Surveys. Throughout both phases of this study you will be asked to complete several different surveys asking about your medical symptoms, exercise, sleep, mood, stress, social support, medical history, medication and supplement intake and other characteristics. While we ask that you answer all questions completely and to the best of your ability, you are free to skip any questions that you would prefer not to answer. Some questionnaires will be completed at the study visits. Others you will complete at home on a secured website (REDCap) 1-3 days before or after study visits. You will receive an email with the subject line "Waves Study Surveys to Complete" on the days you are to complete questionnaires online. The email will contain a link to the surveys. The questionnaires will take less than two hours to complete. If you are unable to complete the questionnaires online, the study team can provide you with paper copies to complete at home. In addition, you will complete the Fatigue Severity Scale and Medical Symptoms Questionnaire on a secured website (REDCap) at home every 4 weeks when you do not have a study visit (weeks 4, 8, 16, 20, 28, 32). You will receive emails with a link to the surveys which will take less than one hour to complete.

Dietary Intake Assessment: weighed food record and food frequency questionnaire. At Visit 1, you will learn how to complete a weighed food record and use the food scale we will loan to you for weighing the amount of food you eat. You will complete food records for 7 consecutive days immediately after Visit 1 and prior to Visits 2, 3 and 4. **Please eat and drink as you normally would during the recording periods.** Do **not** change your food intake. **These food records must be filled out completely with sufficient food details and portion sizes so we can enter the information into our nutrient software program. If you do not do a satisfactory job filling out the food records during the Usual diet phase, the study team can end your participation in the study and you will not move on to the Intervention diet phase. If we have not received a notification that you have mailed back your Food Record, Actigraph and other supplies from visit 1 by day 28 of the study, you will not be able to move on to the Intervention Diet phase of the study.** We will also send a food frequency questionnaire home with you to fill out and bring to your next visit. You will complete a 7 day weighed food record and food frequency questionnaire immediately after Visit 1 and mail it back to us; additional 7-day food records and food frequency questionnaires will be kept immediately prior to Visits 2, 3, and 4 and brought to the study visits.

Physical Activity Assessment: Actigraph. At Visit 1 we will teach you how to use a lightweight (0.5 oz) device (Actigraph) similar to a watch that you will wear on the wrist of your non-dominant hand during each 7-day food recording period. You will wear the Actigraph continuously during the recording periods except when swimming or engaging in other activities where the accelerometer would be submerged in water such as washing dishes or bathing in a tub; you may wear the Actigraph while showering. Any time you remove the Actigraph and put it back on you will document the date and time on forms we will provide. If we do not have at least five days of usable data, including one weekend day, we will ask you to wear the Actigraph for additional days until we have the required data. Because of limited battery life and data storage, the Actigraph will be given at Visit 1 and you will mail it back

with the food records after the first data collection period; the Actigraph will be mailed to you prior to Visit 2, 3, and 4 and you will return it at the study visits. **Please maintain your usual physical activity during the recording period.** Do **not** change your activity. **If you lose the monitor, contact the study coordinator immediately.**

Stool Microbiome Collection (OPTIONAL). One aspect of this study is to analyze what bacteria and other microbes are living in your bowels. At Visit 1, we will train you on the techniques for proper collection and transport of a stool specimen and give you collection supplies. You will collect a stool specimen at home during days 5-7 of the weighed food recording after visit 1 and prior to visits 2, 3, and 4. **That is, you will keep a weighed food record for at least 4 full days before collecting your stool.** You will be asked to complete a paper Stool Collection Questionnaire **the same day you collect your stool**. This questionnaire will take less than one hour to complete. If you have a bowel movement while you are at the study visit, we will ask you to try to collect this sample. At each visit, you will be given the supplies for collecting a stool sample to bring to the study team when you return at the next visit. We prefer that you take no new over the counter vitamins or supplements for one week prior to collecting the stool specimen. The stool sample collected after Visit 1 will be returned by mail along with the food records and Actigraph; stool samples for Visits 2, 3, and 4 will be brought to the study visits. **If you have not had a bowel movement during the collection period days at home or at the study visit we will ask you to take the collection materials home to collect a specimen and then mail it back to us in packaging we will provide. Once you have collected your stool you will begin the intervention diet.**

Please initial below to indicate your preference:
________ I agree to provide stool samples.

________ I do not agree to provide stool samples.

Usual Diet. For the first 83 days (12 weeks) of the study we ask that you continue eating and drinking as you normally do. **Do not change your eating habits or dietary supplements**.
Intervention Diet. The intervention diet begins at Visit 2. You will be randomized to either the low saturated fat (Swank) or the modified paleo (Wahls Elimination) diet group. That means you will have an equal chance, like the flipping of a coin, of being in either group. You will purchase food and prepare your meals at home during the intervention diet phase. In prior studies some participants have reported a 25% increase in their grocery bill while following diets like the study diets, however other participants following diets like the study diets reported saving money because they prepared more meals at home and ate out less frequently.

NOTE -- You should **ONLY** agree to participate in this study if you are confident that **1) you are willing to be randomized into either diet and can adopt and sustain the study diet to which you are randomized and 2) your family is willing to support you in following the study diet.** You may find it helpful to remove from the house foods that are not recommended for the study diet to which you are randomized**.** The foods that will be excluded from the study diet to which you are randomized may include foods that you and your family currently enjoy such as high fat meats, beef and pork, dairy, eggs, or grain based products and you may be encouraged to eat foods that are unfamiliar to you. You will be given a copy of a cookbook relevant to the assigned study diet and other resources to help you

adopt the study diet.

You will be educated on why we believe the study diet is likely to help lower fatigue severity and improve quality of life for people who have multiple sclerosis related fatigue. We will explain why this study is important to do as a family as opposed to as an individual.

You are encouraged to bring your adult companion and family members to study visits, especially Visit 2, so they can learn about the diet. At Visit 2, you and one companion, if present, will be given a sample meal. You will be taught about the assigned study diet and how to use the daily diet checklist which gives you prompts to help you follow the diet successfully. You will be asked to follow your assigned diet specifically and make no other dietary modifications throughout the study so we can maintain the differences between the two study diets. You will meet again with the dietitian at Visits 3 and 4.

Diet Checklists. Once you begin the Intervention phase you will complete a Diet Checklist daily to help you monitor your diet. You will return Diet Checklists to us in a self-addressed, stamped envelope at weeks 16, 20, 28, 32 and bring the checklists to study visits at weeks 24 (Visit 3) and 36 (Visit 4). The dietitian will meet with you to review your diet checklists at Visits 3 and 4.

Coaching Calls. You will receive a coaching call from the dietitian 2-3 days following Visit 2 and then three additional calls approximately one week apart. You will be asked to schedule times for these coaching telephone calls with the dietitian. One additional call will be made after you return the first Diet Checklist around week 16. This will be the last scheduled coaching call, however, you may contact the dietitian at any time to receive support in adopting and maintaining the study diet.

Audio Recording. One aspect of this study involves making audio recordings of your conversation with the dietitian during your in-person diet counseling sessions at Visits 2 and 3. They will be reviewed with the dietitian for quality control and training purposes. The recordings are optional. You will be notified at the visit when the recording will begin. You may cancel this consent at any time during the conversation by asking the dietitian to stop the recording and erase the interview.

Please initial below to indicate your preference:
________ Yes, I give you permission to make audio recording of me during this study.

________ No, I do not give you permission to make audio recording of me during this study.

Supplements and medications. As part of the study diet we will ask you to purchase specific vitamin and mineral supplements to take during the Intervention phase of the study (1 tsp cod liver oil, 1000 mcg methylB12, 5000 IU D3, 1000 mcg methylfolate, one multivitamin/mineral for men over 50 without iron). We will ask you to complete a form that will allow us to reimburse you for supplements you purchased that are on the 'approved' list of vitamins and supplements for the study. You will bring your receipts for any purchases of 'approved' vitamins and supplements to us in order to be reimbursed. We will ask that you continue taking any dietary supplements you were using during the first 12 weeks of the study with the exception of supplements that are similar to those you will be asked to take as part of the study. In that case, you will be asked to discontinue and replace those supplements with study-

approved brands. If your health care practitioner recommended the supplements we are asking you to hold (stop taking) we will with your permission send them a letter notifying them of this so they can determine if it is clinically safe for you to take these supplements along with our study supplements. You will be asked to begin taking the study supplements starting on study day 94 beginning by adding one supplement and then adding another new one every 4th day, that is on study days 94, 97, 100, 103, and 106. This will allow time to determine if there are any side effects. If side effects are noted, stop the supplement and notify study staff immediately. **Besides these five supplements, you should not begin taking any new supplements during the study unless prescribed by your physician.** The target serum vitamin D levels are 50 ng/ml or higher. Serum calcium and vitamin D values will be checked at each visit. If you are not in a healthy range of Vitamin D or calcium your primary care physician will be notified. At each study visit, we ask that you bring all prescription medications and dietary supplements you are taking.

Eye and Associated Measurements (OPTIONAL). Another aspect of this study is to monitor how effectively the brain, central nervous system and eye system can repair itself if given a more optimal environment. To study this, we would like your permission to perform five tests (outlined below) at each visit. These measurements have minimal risk and provide an effective means to monitor disease progression. If you agree to participate in this portion of the study, you may stop participating in the tests at any time during the study. You will need to bring your glasses for both reading and distance for these tasks (if applicable). With your permission, your eye doctor will be contacted to retrieve prescription information and/or old test results. The results from these tests will be used for research purposes only. However, with your permission, we will contact your eye doctor should the tests show any abnormalities that need to be addressed clinically.

<u>Dominant Eye Test.</u> This test will take less than a minute to complete. While both your eyes are open you will point with your index finger exactly at the letter projected on the screen in front of you. The examiner will cover your eyes one at a time to check which eye you see your finger pointed directly at the letter.

<u>Critical Flicker Fusion Test (CFF)</u>. This test will take 10 minutes. You will look into a dark box containing a red light. For the first test, this light will be flickering and the speed of the flickering will increase. Your job will be to indicate when the light no longer appears to be flickering by pushing a button. The test will then be repeated, except this time the red light will be continuous. Your job will be to indicate when the light begins to flicker by pressing a button. The test may be repeated for each eye three times to obtain accurate results.

<u>Optical Coherence Tomography (OCT).</u> This is a non-invasive scan of the eye which will use light beams to produce a picture of your retina. You will undergo OCT scanning of your eyes that might take up to 10 minutes. With your permission, the examiner may use artificial tears to improve your comfort and scan quality. **NO** dilating drops will be given. You will be asked to focus on the small green fixation target without blinking while the machine focuses on your retina. The examiner will then begin the scan. Blinking during the scan is allowed as it will not affect the picture taken, however, blinking can make the procedure take longer. This will be done for each eye. You will be allowed to relax between scans.

Visual Acuity and Contrast Sensitivity Testing
This test is similar to the test you perform in eye clinics. You will be seated 6 feet in front of a screen, and a Number pad will be given to you. The letter "C" will appear on the screen. The letter size will vary, as well as the direction of the gap. Your job will be to indicate which direction the gap in the "C" is pointing by clicking a button. Each eye will be tested individually. The test will be repeated using a fixed size but varying contrast (darkness vs lightness) of the projected "C". This test will take 7 minutes.

RETeval ERG: This test uses a handheld device to test retinal and pupillary response to light. The area over your cheeks will be rubbed with a prep gel to remove skin oils and improve test results, similar to an ECG procedure. A small strip will be adhered beneath your eye and connected to the handheld device (See image to the right). The device will project some flickering lights at your eye during the 5-minute test. You will be asked to focus on a small red light within the device and try **not to smile, blink or talk.** The right eye is tested first then the left. Let the study team know if you have allergies to medical prep gels.

Empatic E4Wristband: You will be given two watch-like devices to wear for the duration of the study visit. The devices record your movement activity, temperature, heart rate and pulse variability. Behave normally while wearing the device; no restrictions of movement and activity are required.

Please initial below to indicate your preference:
________ I agree to eye and associated measurements.

________ I do not agree to eye and associated measurements.

HOW TO PREPARE FOR STUDY VISITS
Exercise--We will ask you to continue any physical activity/exercise regimen you are following at the start of the study and **not to begin any new exercise or physical activity program unless directed to by your physician or physical therapist.**

Before all visits we ask that you avoid strenuous exercise for 24 hours. (Examples of strenuous exercise include: running, weight lifting, swimming, aerobics and other activities that make a person sweat or perspire. Moderate activities such as walking are allowed in the 24 hours before a study visit).

Fasting--We ask that you **fast for 12 hours prior to each study visit**, which means no food or beverages may be consumed other than water. Please **do** drink water while you are fasting. Drawing the blood will be easier if you are well hydrated. You may want to bring a snack to eat after the blood draw is complete. You may also purchase food in the hospital cafeteria.

Prescription Medications, Dietary Supplements, Over-the-Counter Medications – please bring to each study visit bottles of all prescription and over-the-counter medications and supplements you are taking. We ask that you **not** start any new supplements or over-the-counter medications during the study unless directed to by your physician.

WaVeS Study Visit Schedule				
**Visit**	**Length**	**Day**	**Diet**	**Study Activities**
		0	Usual	Fast for 12 hours; no strenuous exercise for 24 hours
1	6 hrs	1	Usual	Consent, vitals, blood, MS Functional Composite, Single digit modalities test , 6 minute walk, eye tests, complete questionnaires; trained on how to collect stool samples, Actigraph, and weighed food records; collect bowel movement if possible
		2	Usual	Weighed food record and Actigraph day 1; Will receive RedCap questionnaires via email to complete, complete these within the next 3 days
		3	Usual	Weighed food record and Actigraph day 2; Complete REDCap questionnaires (if not done)
		4	Usual	Weighed food record and Actigraph day 3 Complete REDCap questionnaires (if not done)
		5	Usual	Weighed food record and Actigraph day 4
		6	Usual	Weighed food record and Actigraph day 5; collect stool sample between now and day 9
		7	Usual	Weighed food record and Actigraph day 6; collect stool sample between now and day 9
		8	Usual	Weighed food record and Actigraph day 7; collect stool sample between now and day 9
		9	Usual	Mail weighed food record, food frequency, stool collection and Actigraph in self-addressed, stamped
				box
		28 (4 wks)	Usual	Complete the FSS and MSQ online via RedCap
		56 (8 wks)	Usual	Complete the FSS and MSQ online via RedCap
		77 (11 wks)	Usual	Weighed food record and Actigraph day 1
		78	Usual	Weighed food record and Actigraph day 2
		79	Usual	Weighed food record and Actigraph day 3
		80	Usual	Weighed food record and Actigraph day 4
		81	Usual	Weighed food record and Actigraph day 5; collect stool sample between now and V2; Will receive RedCap questionnaires via email to complete, complete these before your visit
		82	Usual	Weighed food record and Actigraph day 6; collect stool sample between now and V2; Complete REDCap questionnaires (if not done)
		83	Usual	Weighed food record and Actigraph day 7; collect stool sample between now and V2; Complete REDCap questionnaires (if not done)
2	6 hrs	84 (12 wks)	Interventio n	Return stool collection, food records, food frequency, Actigraph; blood/vitals, MS Functional Composite, Single digit modalities test, 6 minute walk, eye tests, complete questionnaires; Randomized to an Intervention diet, sample meal, diet training with dietitian

		86-87	Intervention	Coaching call with dietitian - #1
		94	Intervention	Coaching call with dietitian - #2
		94	Intervention	Start dietary supplement #1
		97	Intervention	Start dietary supplement #2
		100	Intervention	Start dietary supplement #3
		101	Intervention	Coaching call with dietitian - #3
		103	Intervention	Start dietary supplement #4
		106	Intervention	Start dietary supplement #5
		108	Intervention	Coaching call with dietitian - #4
		112 (16 wks)	Intervention	Mail back diet checklist, complete FSS and MSQ online via RedCap
		118	Intervention	Coaching call with dietitian - #5
		140 (20 wks)	Intervention	Mail back diet checklist, complete FSS and MSQ online via RedCap
		161 (23 wks)	Intervention	Weighed food record and Actigraph day 1
		162	Intervention	Weighed food record and Actigraph day 2
		163	Intervention	Weighed food record and Actigraph day 3
		164	Intervention	Weighed food record and Actigraph day 4
		165	Intervention	Weighed food record and Actigraph day 5; collect stool sample between now and V3; Will receive RedCap questionnaires via email to complete, complete these before your visit
		166	Intervention	Weighed food record and Actigraph day 6; collect stool sample between now and V3; Complete REDCap questionnaires (if not done)
		167	Intervention	Weighed food record and Actigraph day 7; collect stool sample between now and V3; Complete REDCap questionnaires (if not done)
3	4-6 hrs	168 (24 wks)	Intervention	Return stool collection, food records, food frequency, Actigraph; blood/vitals, MS Functional Composite, Single digit modalities test, 6 minute walk, eye tests, complete questionnaires, meet with dietitian
		196 (28 wks)	Intervention	Mail back diet checklist, complete FSS and MSQ online via RedCap

		224 (32 wks)	Interventio n	Mail back diet checklist, complete FSS and MSQ online via RedCap
		245 (35 wks)	Interventio n	Weighed food record and Actigraph day 1
		246	Interventio n	Weighed food record and Actigraph day 2
		247	Interventio n	Weighed food record and Actigraph day 3
		248	Interventio n	Weighed food record and Actigraph day 4
		249	Interventio n	Weighed food record and Actigraph day 5; collect stool sample between now and V4; Will receive RedCap questionnaires via email to complete, complete these before your visit
		250	Interventio n	Weighed food record and Actigraph day 6; collect stool sample between now and V4; Complete REDCap questionnaires (if not done)
		251	Interventio n	Weighed food record and Actigraph day 7; collect stool sample between now and V4; Complete REDCap questionnaires (if not done)
4	4-6 hrs	252 (36 wks)	Interventio n	Return stool collection, food records, food frequency, food scale, Actigraph; blood/vitals, MS Functional Composite, Single digit modalities test, 6 minute walk, eye tests, complete questionnaires, meet with dietitian

Questionnaire Schedule of Events

**Question naires**	**V 1**	**Wk 4**	**Wk 8**	**Sto ol**	**1-3 days Befo re**	**V 2**	**Wk 16**	**Wk 20**	**Sto ol**	**1-3 days Befo re**	**V 3**	**Wk 28**	**Wk 32**	**Sto ol**	**1-3 days Befo re**	**V 4**
FSS	X*	X	X		X		X	X		X		X	X		X	
MSQ	X*	X	X		X		X	X		X		X	X		X	
Health Questions	X*				X					X					X	
Medication Audit	X					X					X					X
Doctor and Emergenc y Contact	X															
Personal Informatio n	X															
MSIS29v2	X*				X					X					X	
Fatigue Scale for Motor and Cognitive Function	X*				X					X					X	
MSQLI MFIS	X*				X					X					X	

MSQLI PDQ	X*				X					X					X	
MSQLI MHI	X*				X					X					X	
IPAQ	X*				X					X					X	
PSQI	X*				X					X					X	
PSS 10	X*				X					X					X	
HADS	X*				X					X					X	
SF36 MSQLI	X*				X					X					X	
Social Support and Eating Habits							X			X					X	
MSQOLI5 4	X*				X					X					X	
Stool Collection Questionn aire				X					X					X		
NHIS Health Practices Survey	X				X					X					X	
Side Effects					X					X					X	
End of Study Survey															X	

*day after visit

Study Visits.

Visit 1 will take approximately 6 hours to complete.
The first visit will occur immediately following signing of this consent. We will have you start drinking water in order to make it easier for staff members to draw your blood. We will measure your vital signs (height, weight, pulse, temperature, blood pressure) and calculate your body mass index (BMI, a measure of your weight and height). If your BMI is below 19, your participation in the study will end for your safety.

If you meet the criteria described above you will begin the Usual diet phase of the study. You will have blood drawn. You will complete physical motor tasks and the eye tests (if you are participating in this part of the study). We will review the medications and dietary supplements you are taking and ask about your health. You will complete questionnaires using a secure online system (REDCap). These questionnaires include information about your demographics data, emergency physician and pharmacy contact information, health issues and symptoms. You will have a functional medicine history interview which is a list of questions about your health from infancy to the current time. You will receive training on how to keep a weighed food record, wear the Actigraph, complete a stool collection (if you are participating in this part of the study) and fill out questionnaires. You will return the food record, food

frequency questionnaire, Actigraph and stool collection (if you are participating in that part of the study) to us by mail immediately after the first recording period is completed on day 9. The Actigraph will be mailed back to you for use during the next recording period immediately prior to Visit 2.

Visit 2 will occur twelve weeks after visit 1 and take approximately 6 hours.
When you arrive at the PIC, we will have you start drinking water in order to make it easier for staff members to draw your blood. You will bring your stool collection (if you are participating in that part of the study), weighed food records, food frequency questionnaire, and Actigraph to the study team who will check them with you for completeness. If questions on the online questionnaires were omitted the study team will ask if you meant to skip the question. If you did not mean to skip the question, that is it was simply overlooked, the study team will review the questions and answers with you to complete the questionnaires. Your weight, temperature, pulse, and blood pressure will be recorded and blood will be drawn. We will review the medications and supplements you are taking and ask about your health. You will complete physical motor tasks and the eye tests (if you are participating in this part of the study). You will be randomized to the study diet, consume a meal that meets study diet guidelines, and meet with the study dietitian. You will be given diet checklist forms to use for the next 12 weeks and two self-addressed stamped envelopes to return the diet checklists. You will be given the supplies for collecting a stool sample to bring to the study team at your return visit if you are participating in this part of the study. You will be given food records to complete 7 days prior to the next visit and a food frequency questionnaire to complete and bring to your next visit. The Actigraph will be mailed to you prior to the recording period.

Visit 3: will occur at 24 weeks and take about 4-6 hours to complete.
When you arrive at the PIC, we will have you start drinking water in order to make it easier for staff members to draw your blood. You will bring your stool collection (if you are participating in that part of the study), weighed food records, food frequency questionnaire, and Actigraph to the study team who will check them with you for completeness. Your weight, blood pressure, temperature, and pulse will be measured. You will have blood drawn. You will complete the physical motor assessments and the eye tests (if you are participating in this part of the study). If questions on the online questionnaires were omitted the study team will ask if you meant to skip the question. If you did not mean to skip the question, that is it was simply overlooked, the study team will review the questions and answers with you to complete the questionnaires. We will review the medications and supplements you are taking and ask about your health. You will meet with the study dietitian to review your Diet Checklists and so you can ask questions and receive support in following the diet. If you are randomized to the Wahls Elimination Diet, the dietitian will discuss with you the procedure for adding nightshades back into your diet if you wish to reintroduce them. You will be given diet checklist forms to use for the next 12 weeks and two self-addressed stamped envelopes to return the diet checklists. You will be given supplies for collecting a stool sample to bring to the study team at your return visit if you are participating in this part of the study. You will be given food records to complete the 7 days prior to the next visit and a food frequency questionnaire to complete and bring to your next visit. The Actigraph will be mailed to you prior to the next recording period.

Visit 4: will occur at 36 weeks and take approximately 4-6 hours complete.
When you arrive at the PIC, we will have you start drinking water in order to make it easier for staff members to draw your blood. You will bring your stool collection (if you are participating in that part of

the study), weighed food records, food frequency questionnaire, and Actigraph to the study team who will check them with you for completeness. You will return the food scale we loaned you. Your weight, blood pressure, temperature, and pulse will be measured. You will have blood drawn. You will complete physical motor tasks and the eye tests (if you are participating in this part of the study). If questions on the online questionnaires were omitted the study team will ask if you meant to skip the question. If you did not mean to skip the question, that is it was simply overlooked, the study team will review the questions and answers with you to complete the questionnaires. We will review the medications and dietary supplements you are taking and ask about your health. You will meet with the study dietitian to review your Diet Checklists and so you can ask questions and receive support in following the diet. You will then view an informational video on how to complete the Functional Medicine Survey, while filling out the survey. Before you leave, you will also watch a thank you video prepared by Dr. Wahls to show our appreciation for your participation in our study.

<u>Use of Email and Text Messaging for Study Communications</u>

Risk of using email and text messaging

Email and text messaging are common ways to communicate. They may also be convenient ways for the participant and researcher to ask questions and receive answers during the research study. Sending information by email or text message, however, has a number of risks that participants should consider prior to use of email and/or texting. These include, but are not limited to:

- Email/text can be circulated, forwarded, stored electronically and on paper, and sent to unintended recipients. They can be intercepted, altered, forwarded, or used without authorization or detection.
- Email/text senders can easily misaddress an email or text sending information to someone other than the intended recipient.
- Backup copies of email/text may exist even after the sender or the recipient has deleted his or her copy.
- Employers and on-line services have a right to inspect email transmitted through their systems.
- Emails/texts can be used as evidence in court.
- Email can be used to introduce viruses into computer systems.
- If your email is a family address, other family members may see your messages

Conditions for use of email and text messaging

The university and researcher cannot guarantee but will use reasonable means to maintain security and confidentiality of email/text information sent and received. The participant and researcher must consent to the following conditions:

1. Email/text is not appropriate for urgent or emergency situations. The researcher cannot guarantee that any particular email/text will be read and responded to within a specific time period.
2. Email/text must be concise. The participant should call or schedule an appointment if the issue is too complex or sensitive to discuss via email/text.
3. The participant should not use email/text for communication regarding sensitive medical information.
4. The researcher will not forward participant-identifiable email/texts outside of the University of Iowa and affiliates without the participant's prior written consent, except as authorized
5. The research team and university are not liable for breaches of confidentiality caused by the participant or any third party.

Please place your initials in the blank next to Yes or No for the question below:
I have read the instructions above regarding use of email and text communications.

_____ Yes   _____ No

Blood/Stool and Study Data Storage for Future Use
As part of this study, we are obtaining **blood and stool and study data** from you. We would like to have the possibility to study your **blood/stool and study data** in the future, after this study is over.

Blood cells removed from the blood samples can be used to make a cell line and DNA. Cell lines are produced by growing blood cells in a laboratory and allow us to have a source of the DNA without having to redraw your blood. These blood cells can be stored for decades or more. The cell lines and DNA could be made available to researchers trying to learn more about the causes of diseases.

The tests we might want to use to study your **blood and stool** may not even exist at this time. Therefore, we are asking for your permission to store your blood and stool data and samples, so that we can study them in the future without contacting you about each new test or study. These future studies may provide additional information that will be helpful in understanding multiple sclerosis, but it is unlikely that what we learn from these studies will have a direct benefit to you. It is possible that your type of blood and stool data might be used to develop products or tests that could be patented and licenses. There are no plans to provide financial compensation to you should this occur.

If you agree now to future use of your **blood/stool and study data**, but decide in the future that you would like to have it removed from future research, you should contact **Dr. Terry Wahls at 319 356 4421.** However, if some research with your **blood/stool and study data** has already been completed, the information from that research may still be used.

Please place your initials in the blank next to Yes or No for the question below.
I will allow my blood and stool and study data may be stored/shared for future research in multiple sclerosis and other health conditions.

________Yes   ______No (initials)

Additional Blood Draw for Storage for Future Use

We would like to draw 9 additional teaspoons of blood from a vein in your arm using a needle. This blood will be drawn at the same time as the other blood is drawn for the study, and would add a maximum of 5 minutes to the blood draw time. This blood will be stored at the University of Iowa Hospitals and Clinics for future testing/research.

If you agree now to the collection and storage of your **blood samples**, but decide in the future that you would like to have it removed from future research, you should contact **Dr. Terry Wahls at 319 356 4421.** However, if some research with your **blood samples** has already been completed, the information from that research may still be used.

Please place your initials in the blank next to Yes or No for the question below.
I will allow additional blood to be drawn and stored for future use.

________Yes ______No (initials)

Genetic Research

One purpose of this study is to look at genes (DNA) and how they affect health and disease. Genes are the instruction manual for the body. The genes you get from your parents decide what you look like and how your body behaves. They can also tell us a person's risk for certain diseases and how they will respond to treatment.

You are being asked to give **blood and stool** samples for genetic research. What we learn about you from this sample will not be put in your health record. No one else (like a relative, boss, or insurance company) will be given your test results. Your test results will not be shared with your doctor. Genetic testing will be done on some of the blood we draw and the stool you collect. **We will not contact you with any specific results from your analysis.**

In order to study your DNA and RNA, blood totaling 3 teaspoons will be drawn from a vein in your arm using a needle if you agree to participate in this portion of the study. This blood will be drawn at the same time as the other blood is drawn for the study, and would add a maximum of 5 minutes to the blood draw time.

Please place your initials in the blank next to Yes or No for the question below.
I will allow extra blood to be drawn for DNA/RNA research.

________Yes ______No (initials)

Genetic Information Nondiscrimination Act (GINA)

A new federal law called the Genetic Information Nondiscrimination Act (GINA) generally makes it illegal for health insurance companies, group health plans, and employers of 15 or more persons to discriminate against you based on your genetic information. Based on this new law, health insurance companies and group health plans are prohibited from requesting your genetic information that we get from this research. This means that they may not use your genetic information when making decisions regarding your eligibility for insurance coverage or the amount of your insurance premiums. Be aware that this new federal law will not protect you against genetic discrimination by companies that sell life insurance, disability insurance, or long-term care insurance. The law also does not prohibit discrimination if you already have a manifest genetic disease or disorder.

Contact for Future Studies

We would like to contact you to invite you to participate in future studies. In order to do this we would keep information on how to reach you (such as mailing address, phone, email) as well as information about your multiple sclerosis diagnosis (such as the type of multiple sclerosis) and general characteristics (such as gender and birthdate). If the answer is yes, you would be contacted and told

about the future studies. Agreeing to participate in this study does not obligate you to participate in any future studies. You would sign a new consent for any future studies.

Please initial below.
I would like to be contacted about future studies.

________Yes ______No

Online Participant Data Storage

Your data for this study will be stored electronically in the REDCap platform. The REDCap platform is managed by the Institute for Clinical and Translational Science at the University of Iowa. Only IRB approved research team members will have access to the REDCap data platform. Each team member will be granted access to the REDCap data system through a secure login. REDCap supports Two-factor authentication using DUO. Two-factor authentication requires something that only the user ***knows***, with a second factor, something only the user ***has*** in their possession, to provide strong identity verification.

In the REDCap data platform, primary data is secured in HCIS Pomerantz Data Center. Data backups are secured in ITS Lindquist Data Center. Operating system security includes: secure logins, data encryption at rest, remote system logging and configuration and change management. Data backups are encrypted both in flight and at rest. Copies of data are replicated to the remote data center every 15 minutes. There are 100+ points in time copies of data available at any time. Disaster recovery has been tested. You will have the option to complete some study questionnaires online via REDCap rather than on paper if you prefer to do so.

Confidentiality of study procedures and study materials

In order to protect the integrity of the study it is important that you **not** share the study materials and procedures with others outside of your household. Please do not share study materials and procedures on social media, internet sites or in any way copy them or send them to others.

WHAT ARE THE RISKS OF THIS STUDY?

You may experience one or more of the risks below from being in this study. In addition to these, there may be other unknown risks, or risks that we did not anticipate, associated with being in this study.

There may be a loss of confidentiality.

Cognitive Decline

As with any person with MS, it is possible that you will experience some cognitive decline. If we notice that you have experienced cognitive decline throughout the study, we will ask you to retake the Short Portable Mental Status Questionnaire. If your score has decreased since screening or if your score now falls into the moderate to severe cognitive impairment categories, we will recommend that you visit your treating neurologist. We will also send your neurologist a letter which explains that we have noticed a cognitive decline. This letter will ask your neurologist to determine if you are cognitively suitable to continue participating in the study. You will be able to continue with the study if your neurologist deems it okay. If your neurologist deems you to be unable to continue in the study, the research team will end

your participation.

Surveys
Filling out surveys is usually low risk. You may be tired or embarrassed if you find the surveys difficult to complete. We will watch your level of fatigue and give you opportunities to rest or reschedule to complete the tests. You may skip any question you do not want to answer.

Nutrition
Following the nutritional advice to implement the study diet is expected to be low risk. Following the advice will likely cause weight loss, or more unlikely, weight gain. If you lose too much weight we will ask you to weight yourself at home once each week for two weeks (in the morning after urinating and wearing typical street clothes) and report that weight to the study team. If your weight continues to remain low we will ask you to follow up with your personal physician. We will also ask the study dietitian to contact you by phone to help you increase your calorie intake by suggesting foods that are compliant with the diet you have been assigned. For example, we will ask you to consume more meat and fats such as nuts and coconut oil if you are assigned to the Wahls Elimination Diet, or white poultry and fish if you are assigned to the Swank Diet. You will continue weighing yourself once a week and reporting your weight to the study team. When you weight reaches a safe level you can discontinue the weekly weights

You may miss eating foods that are excluded from the study diet. It is possible that you will be asked to eat foods whose taste you do not enjoy at this time. It is also possible, although unlikely, that the food may have an interaction with medicines you are taking. Following the nutritional advice may increase the cost of your weekly grocery bill. Eating meals prepared at home will reduce the expense of implementing the study diet. It is hard to know how the new foods and supplements you will be taking will affect your medications. Those taking Coumadin (warfarin) are not allowed to participate in the study.

It is also possible that your disease will continue to progress and your various (or new) multiple sclerosis related symptoms will worsen (or develop) while on the study diet.

Because we are informing you of the potential connection between the foods you eat and MS related symptoms, and the potential effects of food on physical symptoms in general, your awareness of new physical symptoms might be heightened by participation in this study. These symptoms may include, but are not limited to, a rash, transient headaches, or muscle or joint pain, which are self-limited and would be expected to resolve within a week.

Other side effects of the diets may include:
- Diarrhea, nausea, bloating and other abdominal complaints
- Increased risk of certain kinds of kidney stones.
- Foods on the study diet may be more expensive, foods that are recommended may not be familiar and as tasty; foods that are excluded may be familiar comfort foods that are missed.
- It will take more time to prepare home cooked meals.

Coconut oil risk (Wahls Elimination Diet only)
- Theoretic increase in total cholesterol

- Rare cases of severe allergic reaction

Caffeine restriction risk (Swank Diet only)
- May experience headaches if your intake of coffee/tea needs to be reduced so as not to exceed 3 cups/day

Methyl folate
- May increase risk of cancer
- May increase risk of cardiovascular events
- May produce a bad taste in the mouth; confusion and irritability

Methyl B12
- Diarrhea

Cod Liver Oil
- May increase the risk of vitamin A and D toxicity if taken at levels much higher than used in the study confusion or unusual excitement; diarrhea; dizziness or drowsiness; double vision; severe headache, irritability, or vomiting; and peeling of skin, especially on the lips or palms.

Vitamin D
- May increase the risk of vitamin D toxicity. This could include development of kidney stones or kidney failure, hypertension, and mental changes.

Multivitamin/multimineral without iron
- May cause nausea, vomiting and or diarrhea

Blood Collection
- Collection of blood samples may cause bruising or bleeding at the site of the blood draw.
- There could be some “pin-prick” discomfort from the needle used to draw the blood.

Motor Assessments (walk, hand coordination, symbol digit tasks)
- You may feel embarrassed about your physical abilities if you find the tasks difficult to complete.

Actigraph
- Wearing the Actigraph may cause skin irritation around your wrist.

Bowel movement collection
- Collection of bowel movement samples may be embarrassing and inconvenient to complete. However, you can do this in the privacy of your home and we will provide plastic gloves that you can wear when making the collection.

Critical Flicker Fusion (CFF)
- You may get tired or your eyes may fatigue due to completing the tests. You will be given breaks and encouraged to blink your eyes between tests.

Optical Coherence Tomography (OCT)

- This typically does not carry any risk. Fatigue is possible when a patient is having additional scans performed.

Visual Acuity and Contrast Sensitivity Testing using the Freiburg Vision Test (FrACT)

- You may feel fatigued. You will be given the time to rest between tests.

RETeval

- Irritation related to using the wipe to your eyelids may occur and usually subsides spontaneously.

Empatic E4Wristband

- Minimal thermal emission and LED light exposure. However, it is within daily exposure limits.

Genetic Research

- One risk of giving samples for this research may be the release of your name that could link you to the stored samples and/or the results of the tests run on your samples. To prevent this, these samples will be given a code. Only the study staff will know the code. The name that belongs to the code will be kept in a locked file or in a computer with a password. Only Cathy Chenard, Drs. Snetselaar and Wahls and other approved team members will have access to your name.

WHAT ARE THE BENEFITS OF THIS STUDY?

You may not benefit from being in this study. However, we hope that, in the future, other people might benefit from this study because it will help determine if this dietary approach is effective in treating multiple sclerosis related fatigue.

WHAT OTHER TREATMENT OPTIONS ARE THERE?

Before you decide whether to be in this study, your doctor will discuss the other options that are available to you. The diet in this study is in addition to whatever treatments your doctor has already prescribed. You will continue to take any medications your doctor has prescribed throughout the study.

WILL IT COST ME ANYTHING TO BE IN THIS STUDY?

You will have costs for being in this research study. Reimbursement for some study expenses will be provided. You will have travel and transportation costs and parking costs if you park a car in the parking ramps near the University of Iowa Hospital and Clinics.
You will **not** be reimbursed for meals, incidentals or food costs. You may have a higher food bill as you adopt the study diet. The higher cost may be offset by preparing more home cooked meals.

You will be advised to begin two B vitamins, methyl folate and methyl B12, cod liver oil, and a multivitamin/multimineral without iron as part of the study diet. If your vitamin D level is low, we will ask you to begin vitamin D and follow up with your personal physician to correct low levels of vitamin D.

WILL I BE PAID FOR PARTICIPATING?

You will not be paid money for being in this research study. You will, however, receive a cookbook that

is relevant to your study diet to which you are assigned.

The study may reimburse you for your mileage to and from study visits according to the published IRS reimbursement rate for medical visits up to a maximum of $288 per visit. If you park in the hospital ramp we will provide hourly parking passes to cover the cost of parking your car during the time of the study visits. If you will travel more than 140 miles to the study visit (280 miles round trip), you can be reimbursed for one night of lodging per study visit up to $89 per visit if you provide us with an itemized hotel/motel bill/folio. Lodging reimbursement is limited to the actual/reasonable cost of a single room plus applicable taxes. Travelers are encouraged to ask for the discounted rate when making reservations (i.e., the government/educational/corporate rate). We will provide you with a list of local hotels and motels.

You may be reimbursed for the costs of the study dietary supplements if you purchase the supplement brands that are on the approved list and provide your receipts**.** Shipping costs associated with supplement purchase will <u>not</u> be reimbursed. You will be asked to complete a form that includes your social security number and your banking information so that you can be reimbursed. You may elect to not provide that information but then you would not be reimbursed for the cost of the various study supplements. You will have the option to complete an Authorization for Electronic Payments form that includes your social security number and your banking information if you wish to have the reimbursement directly deposited into your bank account rather than have a check mailed to you.

DO THE RESEARCHERS HAVE PERSONAL FINANCIAL INTEREST IN THIS STUDY?

One of the study team members has reported a personal financial interest related to this project. Dr. Terry Wahls has disclosed that she has received royalties from the book, The Wahls Protocol that is given to individuals participating in this study.

The University's Conflict of Interest in Research Committee has reviewed Dr. Wahls' financial interest and developed a plan to manage it. The management plan requires that you be informed of the financial interest in this consent form for the study.

WHO IS FUNDING THIS STUDY?

The study is being funded by the National Multiple Sclerosis Society (NMSS) and the University of Iowa Foundation. No one on the research team will receive a direct payment or increase in salary from the National Multiple Sclerosis Society (NMSS) and the University of Iowa Foundation for conducting this study.

WHAT IF I AM INJURED AS A RESULT OF THIS STUDY?

If you are injured or become ill from taking part in this study, medical treatment is available at the University of Iowa Hospitals and Clinics.

The University of Iowa does not plan to provide free medical care or payment for treatment of any illness or injury resulting from this study unless it is the direct result of proven negligence by a University employee.

If you experience a research-related illness or injury, you and/or your medical or hospital insurance

carrier will be responsible for the cost of treatment.

WHAT ABOUT CONFIDENTIALITY?

We will keep your participation in this research study confidential to the extent permitted by law. However, it is possible that other people such as those indicated below may become aware of your participation in this study and may inspect and copy records pertaining to this research. Some of these records could contain information that personally identifies you.

- federal government regulatory agencies,
- auditing departments of the University of Iowa, and
- the University of Iowa Institutional Review Board (a committee that reviews and approves research studies)

To help protect your confidentiality, we will use a study ID and not your name on data collection materials. The file that connects your name and Study ID will be kept in a locked room. Your consents and materials with your name will be kept in a locked room. The electronic data will be kept in the university internal medicine network folder. Network access will be limited to only approved members of the study team. Each study team will access the folder through a secure login. REDCap data entry and storage of participant data for this study will be stored electronically in the REDCap platform. The REDCap platform is managed by the Institute for Clinical and Translational Science at the University of Iowa. Only IRB approved research team members will have access to the REDCap data platform. Each team member will be granted access to the REDCap data system through a secure login.

If we write a report or article about this study or share the study data set with others, we will do so in such a way that you cannot be directly identified.

The University of Iowa Hospitals and Clinics generally requires that we document in your medical record chart that you are participating in this study. The information included in the chart will provide contact information for the research team as well as information about the risks associated with this study. We will keep this Informed Consent Document in our research files; it will not be placed in your medical record chart.

WILL MY HEALTH INFORMATION BE USED DURING THIS STUDY?

The Federal Health Insurance Portability and Accountability Act (HIPAA) requires University of Iowa Health Care to obtain your permission for the research team to access or create "protected health information" about you for purposes of this research study. Protected health information is information that personally identifies you and relates to your past, present, or future physical or mental health condition or care. We will access or create health information about you, as described in this document, for purposes of this research study. Once University of Iowa Health Care has disclosed your protected health information to us, it may no longer be protected by the Federal HIPAA privacy regulations, but we will continue to protect your confidentiality as described under "Confidentiality."

We may share your health information related to this study with other parties including federal government regulatory agencies, the University of Iowa Institutional Review Boards and support staff.

You cannot participate in this study unless you permit us to use your protected health information. If you choose *not* to allow us to use your protected health information, we will discuss any non-research

alternatives available to you. Your decision will not affect your right to medical care that is not research-related. Your signature on this Consent Document authorizes University of Iowa Health Care to give us permission to use or create health information about you.

Although you may not be allowed to see study information until after this study is over, you may be given access to your health care records by contacting your health care provider. Your permission for us to access or create protected health information about you for purposes of this study has no expiration date. You may withdraw your permission for us to use your health information for this research study by sending a written notice to **Terry Wahls MD** at **200 Hawkins Drive SE636 GH, Iowa City, Iowa 52242**. However, we may still use your health information that was collected before withdrawing your permission. Also, if we have sent your health information to a third party, such as the study sponsor, or we have removed your identifying information, it may not be possible to prevent its future use. You will receive a copy of this signed document.

IS BEING IN THIS STUDY VOLUNTARY?

Both the Usual diet phase and the Intervention diet phase are completely voluntary. You may choose not to take part at all. If you decide to be in this study, you may stop participating at any time. If you decide not to be in this study, or if you stop participating at any time, you won't be penalized or lose any benefits for which you otherwise qualify.

What if I Decide to Drop Out of the Study? If you decide to leave the study early, we will ask you to tell us why you have chosen to leave the study before completing the entire study and ask if you would complete all of the surveys and forms so we have as much data as possible about your participation. If you are willing to come for an in person visit we will also complete the physical assessments as well. If you are not able to return in person we would still like very much to receive the diet checklists, Actigraph and completed questionnaires. If you have lost your copies of questionnaires we will mail a fresh copy of them to you and include a return prepaid shipping envelope.

Will I Receive New Information About the Study while Participating?

If we obtain any new information during this study that might affect your willingness to continue participating in the study, we'll promptly provide you with that information.

Can Someone Else End my Participation in this Study?

Under certain circumstances, the study team might decide to end your participation in this research study earlier than planned. This might happen because in the judgment of the study team it would not be safe for you to continue in the study or because funding for the study has ended. If you are not able or willing to provide blood samples, if you do not do a satisfactory job keeping the weighed food records during the Usual diet phase the study team may end your participation at that time. Because both the Usual diet phase (12 weeks) and the Intervention diet phase are relatively brief (only 24 weeks) it is highly unlikely that this would occur.

WHAT IF I HAVE QUESTIONS?

We encourage you to ask questions. If you have any questions about the research study itself, please contact: the clinical coordinator, Mary Ehlinger at 319-384-5002. You can also contact a member of the study team by emailing MSDietStudy@healthcare.uiowa.edu. If you experience a research-related

injury, please contact: Dr. Terry Wahls at 319-356-4421. If you have questions, concerns, or complaints about your rights as a research subject or about research related injury, please contact the Human Subjects Office, 105 Hardin Library for the Health Sciences, 600 Newton Rd, The University of Iowa, Iowa City, IA 52242-1098, 319-335-6564, or e-mail irb@uiowa.edu. General information about being a research subject can be found by clicking "Info for Public" on the Human Subjects Office web site, http://hso.research.uiowa.edu/. To offer input about your experiences as a research subject or to speak to someone other than the research staff, call the Human Subjects Office at the number above.

This Informed Consent Document is not a contract. It is a written explanation of what will happen during the study if you decide to participate. You are not waiving any legal rights by signing this Informed Consent Document. Your signature indicates that this research study has been explained to you, that your questions have been answered, and that you agree to take part in this study. You will receive a copy of this form.

Subject's Name (printed): ____

Do not sign this form if today's date is on or after EXPIRATION DATE: 06/08/18 .

____ ______________________________

(Signature of Subject) (Date)

Statement of Person Who Obtained Consent

I have discussed the above points with the subject or, where appropriate, with the subject's legally authorized representative. It is my opinion that the subject understands the risks, benefits, and procedures involved with participation in this research study.

____ ______________________________

(Signature of Person who Obtained Consent) (Date)
